# Peripheral cathepsin L inhibition induces fat loss in *C. elegans* and mice through promoting central serotonin synthesis

**DOI:** 10.1186/s12915-019-0719-4

**Published:** 2019-11-26

**Authors:** Yan Lin, Bin Bao, Hao Yin, Xin Wang, Airong Feng, Lin Zhao, Xianqi Nie, Nan Yang, Guo-Ping Shi, Jian Liu

**Affiliations:** 1 School of Food and Biological Engineering, Hefei University of Technology, 193 Tunxi Road, Hefei, 230009 Anhui China; 2 Hefei National Laboratory for Physical Sciences at Microscale, University of Science and Technology of China, Hefei, 230026 Anhui China; 3 Department of Medicine, Brigham and Women’s Hospital and Harvard Medical School, Boston, MA 02115 USA; 4 Engineering Research Center of Bio-process, Ministry of Education, Hefei University of Technology, Hefei, 230009 China

**Keywords:** Peripheral cathepsin L, Central serotonin, Fat accumulation, *C. elegans*, Mice

## Abstract

**Background:**

Cathepsin L and some other cathepsins have been implicated in the development of obesity in humans and mice. The functional inactivation of the proteases reduces fat accumulation during mammalian adipocyte differentiation. However, beyond degrading extracellular matrix protein fibronectin, the molecular mechanisms by which cathepsins control fat accumulation remain unclear. We now provide evidence from *Caenorhabditis elegans* and mouse models to suggest a conserved regulatory circuit in which peripheral cathepsin L inhibition lowers fat accumulation through promoting central serotonin synthesis.

**Results:**

We established a *C. elegans* model of fat accumulation using dietary supplementation with glucose and palmitic acid. We found that nutrient supplementation elevated fat storage in *C. elegans*, and along with worm fat accumulation, an increase in the expression of *cpl-1* was detected using real-time PCR and western blot. The functional inactivation of *cpl-1* reduced fat storage in *C. elegans* through activating serotonin signaling. Further, knockdown of *cpl-1* in the intestine and hypodermis promoted serotonin synthesis in worm ADF neurons and induced body fat loss in *C. elegans* via central serotonin signaling. We found a similar regulatory circuit in high-fat diet-fed mice. Cathepsin L knockout promoted fat loss and central serotonin synthesis. Intraperitoneal injection of the cathepsin L inhibitor CLIK195 similarly reduced body weight gain and white adipose tissue (WAT) adipogenesis, while elevating brain serotonin level and WAT lipolysis and fatty acid β-oxidation. These effects of inhibiting cathepsin L were abolished by intracranial injection of p-chlorophenylalanine, inhibitor of a rate-limiting enzyme for serotonin synthesis.

**Conclusion:**

This study reveals a previously undescribed molecular mechanism by which peripheral CPL-1/cathepsin L inhibition induces fat loss in *C. elegans* and mice through promoting central serotonin signaling.

## Background

Lysosomal cathepsins play an important role in various physiological processes and diseases, such as adaptive immunity, rheumatoid arthritis, cardiovascular diseases, and cancer [1]. Over the past 15 years, some of the proteinases have been implicated in the development of obesity through controlling adipogenesis. The circulating levels of cathepsin L and S, which correlate significantly with body weight indexes, significantly increase in clinical obese individuals [2–4]. Obese humans and mice have higher expressions of cathepsin D, K, L, and S in white adipose tissues (WAT) than lean controls [2, 3, 5–8]. In obese patients and/or mice undergoing weight loss, the reduction in body weight is accompanied by the decline in WAT and circulating levels of cathepsin K, L, and S [3, 4, 6, 8]. In vitro studies show that the expressions of cathepsin D, K, L, and S are elevated along with fat accumulation during human and murine adipocyte differentiation, and cathepsin inhibition can reduce adipogenesis of the cells [2, 5, 7, 9, 10]. In contrast, cathepsin L and K overexpression in 3T3-L1 pre-adipocytes and the treatment of pre-adipocytes with human recombinant cathepsin S enhance adipogenesis [2, 9, 10]. Utilizing genetic deficiency and pharmacological inhibition of cathepsin K and cathepsin L, our previous studies demonstrate their essential roles in mouse adipogenesis and body weight gain and attribute the actions to the degradation of extracellular matrix protein fibronectin [2, 9]. However, beyond degrading fibronectin, the detailed molecular mechanisms that cathepsins control fat accumulation remain unclear.

Over the past decade, *Caenorhabditis elegans* has emerged as a powerful animal model for exploring the molecular mechanism of fat metabolism and obesity [11–13]. As the first wholly sequenced multicellular organism, *C. elegans* shares at least 83% of its protein sequences with humans [12] and over 70% of its lipid genes have human orthologs [14]. Most importantly, its core lipid metabolic pathways, such as insulin, TOR, serotonin, dopamine, and glutamate pathways, are highly conserved with mammals [11]. Moreover, genetic tractability and transparent bodies of *C. elegans* make it highly suitable for investigating the complicated molecular pathways and tissue interaction with respect to fat metabolism. The possible involvement of worm cathepsin-like protein in fat storage in *C. elegans* remains untested.

In this study, we firstly established a *C. elegans* model of fat accumulation and utilized it to uncover that the expression of cathepsin L-like protease CPL-1 increased along with nutrient-induced fat accumulation in *C. elegans*. Moreover, we found that mutation and knockdown of *cpl-1* reduced fat storage in *C. elegans*. Further, a feedback circuit, which CPL-1 inhibition in the intestine and hypodermis promoted central serotonin synthesis to induce fat loss, was demonstrated in *C. elegans*. Finally, this conserved circuit, linking the peripheral fat storage tissue and the central nervous system, was also confirmed in mice fed with a high-fat diet (HFD).

## Results

### The supplementation of glucose or palmitic acid elevated fat storage and the expression of cathepsin L-like protease CPL-1 in *C. elegans*

**Fig. 1:**
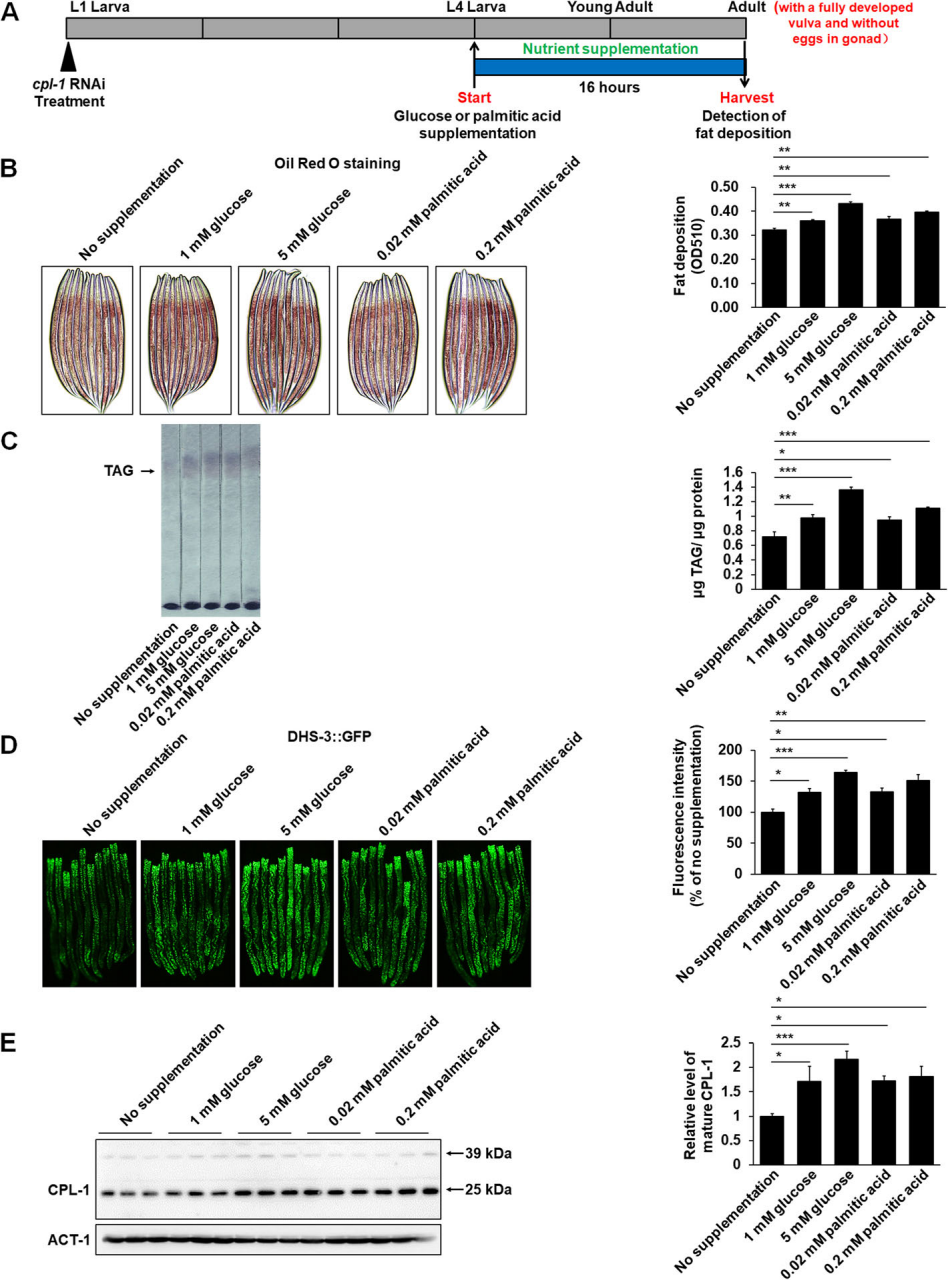
The supplementation of nutrients elevated fat storage and the expression of CPL-1 in *C. elegans*. **a** Schematic representation of glucose or palmitic acid supplementation. **b** Representative images and quantification of Oil Red O staining in N2 worms induced by the supplementation of glucose (at 1 mM and 5 mM) or palmitic acid (at 0.02 mM and 0.2 mM). For Oil Red O staining quantifications, the data were obtained from 3 independent experiments and 3000 stained worms were used in each experiment. **c** Representative image of thin-layer chromatography (TLC) and triglyceride (TAG) contents in N2 worms induced by the supplementation of glucose or palmitic acid, *n* = 3 independent growths. **d** Representative images and quantification of GFP fluorescence in DHS-3::GFP worms induced by the supplementation of glucose or palmitic acid. For GFP fluorescence quantification, the data were obtained from 3 independent experiments and 30 worms were imaged and qualified with the level of fluorescence intensity. **e** Immunoblot and quantification of CPL-1 protein in N2 worms induced by the supplementation of glucose or palmitic acid. The band of mature protein at 25 kD, the active form of CPL-1, was counted as the quantification of CPL-1 to ACT-1, *n* = 3 independent growths. The data in **b**–**e** are presented as mean ± SEM. *p < 0.05; **p < 0.01; ***p < 0.001. n.s. not significant by one-way ANOVA

In the laboratory, *C. elegans* is usually grown on Nematode Growth Medium (NGM) plates seeded with *Escherichia coli* OP50. To establish a model of nutrient-induced fat deposition in *C. elegans*, we added glucose (at 1 mM and 5 mM) or palmitic acid (at 0.02 mM and 0.2 mM) into the NGM plates. As shown in Fig. 1a, the synchronized worms were grown on standard NGM plates seeded with OP50. At the fourth larval (L4) stage, they were transferred onto nutrient-supplementing NGM plates seeded with OP50. After 16 h, the adult worms as judged by the presence of fully developed vulvas were harvested. Under these conditions, the supplementation of the nutrients gradually increased the levels of glucose (Additional file 1: Figure S1A and S1B) and palmitic acid (Additional file 1: Figure S1C and S1D) in both *E. coli* and *C. elegans*, but they did not affect the developmental rates of worms (Additional file 2: Table S1). Utilizing Oil Red O staining to detect worm neutral triglycerides (TAG), we found that the supplementation of the nutrients increased fat accumulation in wild-type N2 worms (Fig. 1b). Further, using dye-independent thin-layer chromatography (TLC) analysis and the worms expressing lipid droplet marker DHS-3::GFP [15], we demonstrated that the supplementation of the nutrients elevated TAG content in N2 worms (Fig. 1c) and enhanced green fluorescence intensity (Fig. 1d), GFP expressions (Additional file 3: Figure S2A), and the size of lipid droplets (Additional file 3: Figure S2B and S2C) in DHS-3::GFP worms. The results affirmed the enhancement in worm fat storage after the supplementation of the nutrients. Thus, a *C. elegans* model of fat accumulation is established.

During mammalian preadipocyte differentiation, the expressions of multiple cathepsins are also increased along with fat accumulation [9]. To understand whether their homologous proteases were involved in nutrient-induced fat accumulation in *C. elegans*, we used real-time PCR to detect the expressions of candidate cathepsin-like genes in N2 worms grown on standard NGM plates and NGM plates with 5 mM glucose. As shown in Additional file 4: Table S2, 5-mM glucose supplementation significantly affected the expression of six genes, including *cpr-1(C52E4.1)*, *cpr-4(F44C4.4)*, *cpr-5(W07B8.5)*, *cpr-6(C25B8.3)*, *asp-12(F21F8.4)*, and *cpl-1(T03E6.7)*. And *cpl-1*(*T03E6.7*) demonstrated the highest induction in response to 5-mM glucose supplementation (Additional file 5: Figure S3A and Additional file 4: Table S2). Further, we examined the expressions of these six genes after glucose (at 1 mM and 5 mM) or palmitic acid (at 0.02 mM and 0.2 mM) treatments. Among the genes, only the expression of *cpl-1(T03E6.7)* was significantly elevated by nutrient supplementation in a dose-dependent manner (Additional file 5: Figure S3B). To examine the changes of mature (at 25 kD) and pro-enzyme (at 39 kD) protein of CPL-1, we further performed western blot analysis using anti-CPL-1 polyclonal antibodies. Supporting the results in real-time PCR analysis (Additional file 5: Figure S3 and Additional file 4: Table S2), the supplementation of glucose and palmitic acid also significantly elevated the levels of mature CPL-1 in a dose-dependent manner (Fig. 1e). The results suggest a substantial role of CPL-1 in fat accumulation in *C. elegans*.

### Functional inactivation of CPL-1 suppressed fat accumulation in *C. elegans*

**Fig. 2:**
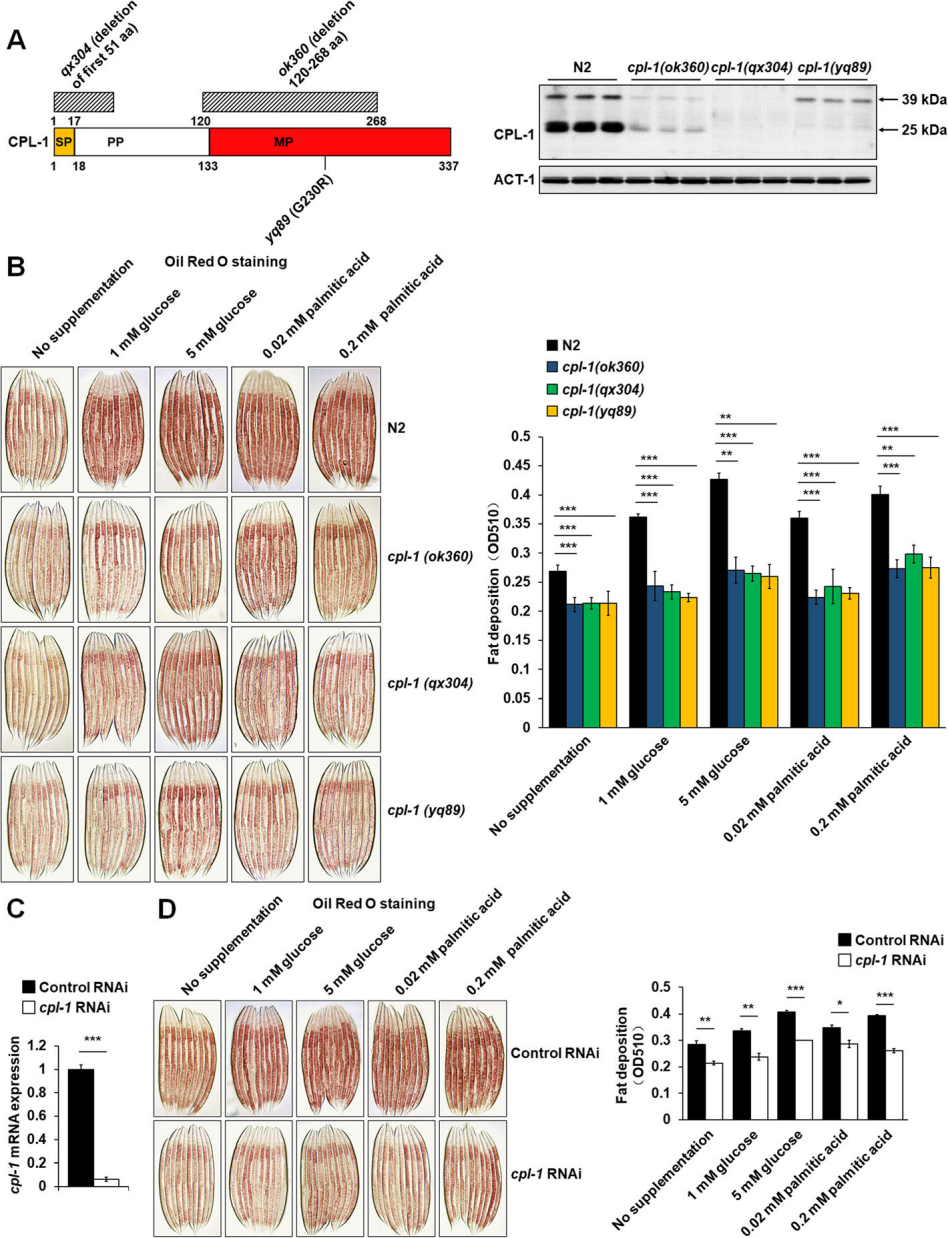
Functional inactivation of CPL-1 suppressed fat accumulation in *C. elegans.* **a** Schematic representation of *C. elegans* CPL-1 and the immunoblot for CPL-1 protein in three *cpl-1* mutants *ok360*, *qx304*, and *yq89*. SP, signal peptide; PP, propeptide; MP, mature peptide. **b** The representative images and quantification of Oil Red O staining in N2 and *cpl-1*-mutant worms induced by supplementation of glucose or palmitic acid. **c** Real-time PCR analysis of the expression of *cpl-1* in control or *cpl-1* knockdown worms, *n* = 3 independent growths. *act-1* was used as the reference gene in real-time PCR analysis. **d** The representative images and quantification of Oil Red O staining in control or *cpl-1* knockdown worms induced by supplementation of glucose or palmitic acid. For Oil Red O staining quantifications, the data were obtained from 3 independent experiments and 3000 stained worms were used in each experiment. The data in **b**–**d** are presented as mean ± SEM; for **b**, two-way ANOVA; and for **c**, **d**, two-tailed Student’s *t* test was used for statistical analysis. *p < 0.05; **p < 0.01; ***p < 0.001

To assess the possible effect of CPL-1 activity on worm fat storage, we studied the developmental rates and fat content of three *cpl-1* mutant alleles *ok360*, *qx304*, and *yq89*. As shown in Fig. 2a, *ok360* contains a deletion of 120–268 amino acids in CPL-1 protein sequence, and *qx304* lacks the first 51 amino acids, and *yq89* has a G230R mutation in the Pept_C1 domain in CPL-1 protein [16]. As reported previously [16, 17], the *cpl-1* mutants normally developed when cultured at 20 °C (Additional file 6: Table S3). However, Oil Red O staining showed that body fat accumulations of the *cpl-1* mutants were obviously lower than those of N2 worms, which grew on either standard NGM plates or nutrient-supplementing NGM plates (Fig. 2b). Further, RNAi-mediated inactivation of *cpl-1* was performed in N2 worms (Fig. 2c and Additional file 9: Figure S5A). Although knockdown of *cpl-1* did not affect the worm developmental rates (Additional file 7: Table S4), Oil Red O staining demonstrated that the *cpl-1* knockdown worm had less basal and nutrient-induced fat storage (Fig. 2d). Finally, using the DHS-3::GFP worms and TLC analysis, we confirmed the effects of the *cpl-1* mutation (Additional file 8: Figure S4A and S4B) and knockdown (Additional file 8: Figure S4C and S4D) on worm fat accumulation. Thus, inhibiting CPL-1 activity with a genetic mutation or RNA silencing induces fat loss in *C. elegans*.

### Functional inactivation of CPL-1 reduced fat storage through activating serotonin signaling in *C. elegans*

**Fig. 3:**
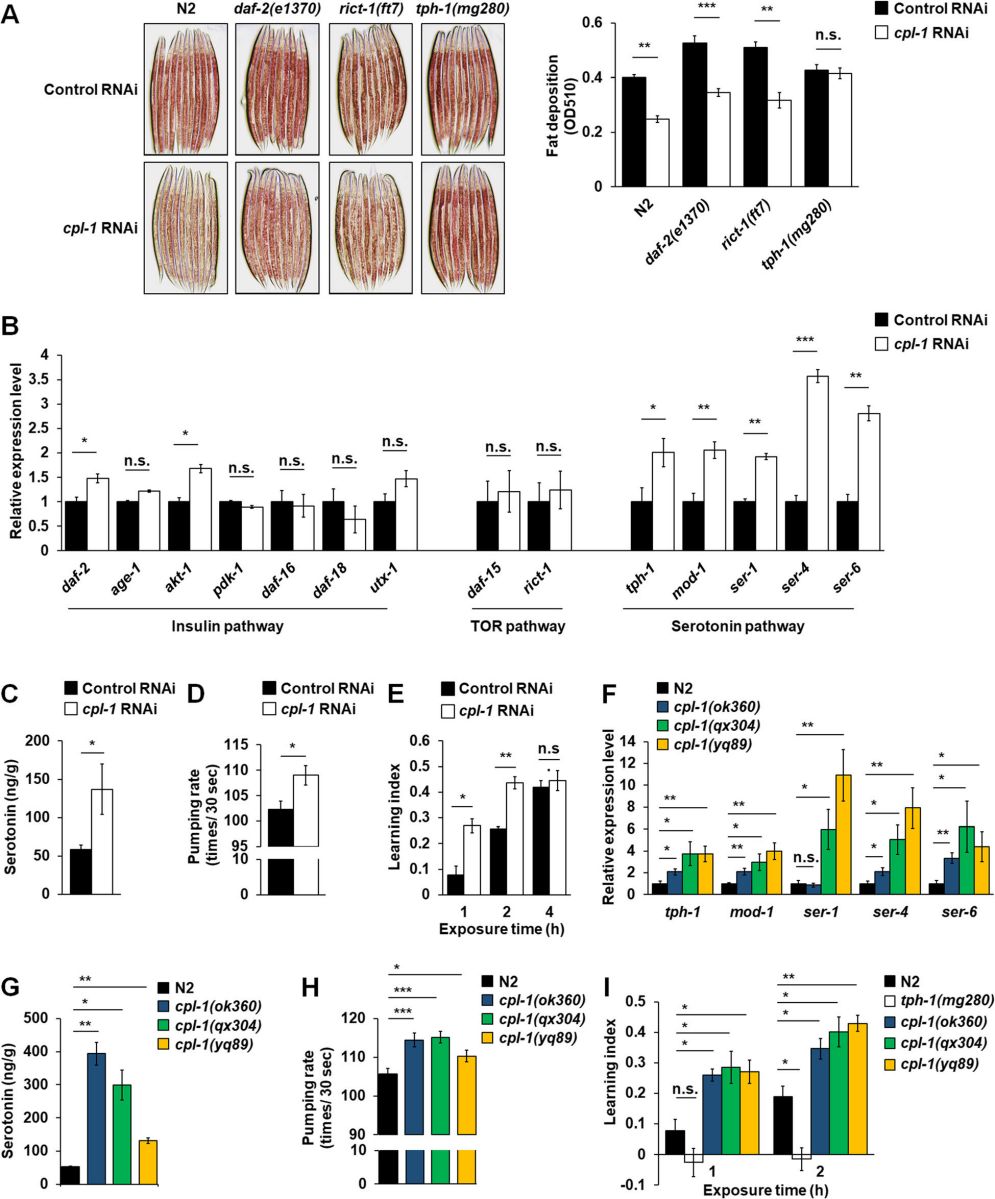
Functional inactivation of CPL-1 reduced fat storage through activating serotonin signaling in *C. elegans*. **a** Representative images and quantification of Oil Red O staining in N2, *daf-2(e1370)*, *rict-1(ft7)*, and *tph-1(mg280)* worms fed with control or *cpl-1* RNAi bacteria. The data were obtained from 3 independent experiments, and 3000 stained worms were used in each experiment. **b** Real-time PCR analysis of genes related to insulin, TOR, and serotonin signaling in N2 worms fed with control or *cpl-1* RNAi bacteria. *act-1* was used as the reference gene in real-time PCR analysis, *n* = 3 independent growths. **c** The serotonin levels in N2 worms fed with control or *cpl-1* RNAi bacteria; the data were based on 3 independent experiments. **d** The pharyngeal pumping rate in N2 worms fed with control or *cpl-1* RNAi bacteria; the data were obtained from 3 independent experiments, and 30 worms were used in each experiment. **e** The two-choice olfactory preference assays to PA14 and HT115 bacteria in N2 worms fed with control or *cpl-1* RNAi bacteria, *n* = 3 independent growths. **f** Real-time PCR analysis of genes involved in the serotonin pathway in N2 worms and *cpl-1* mutants. *act-1* was used as the reference gene in real-time PCR analysis, *n* = 3 independent growths. **g** The serotonin levels in N2 worms and *cpl-1* mutants; the data were based on 3 independent experiments. **h** The pharyngeal pumping rate in N2 worms and *cpl-1* mutants; the data were obtained from 3 independent experiments, and 30 worms were used in each experiment. **i** The two-choice olfactory preference assays to PA14 and OP50 bacteria in N2 worms and *cpl-1* mutants, *n* = 3 independent growths. All data are presented as mean ± SEM. For **a**–**e**, two-tailed Student’s *t* test; for **f**–**i**, one-way ANOVA was used for statistical analysis. *p < 0.05; **p < 0.01; ***p < 0.001. n.s., not significant

In *C. elegans*, some conserved signaling pathways, such as insulin, TOR, and serotonin pathways, have been reported to regulate body fat [11, 18]. Here, to assess possible involvement of the molecular pathways in CPL-1 inhibition-mediated fat loss, we performed RNAi-mediated inactivation of *cpl-1* in *daf-2* (insulin signaling), *rict-1* (TOR signaling), and *tph-1* (serotonin signaling) mutant strains. Similar to those of N2 worms, knockdown of *cpl-1* did not affect the development rates of the mutants (Additional file 7: Table S4), but dramatically depressed mRNA (Additional file 9: Figure S5B) and protein (Additional file 9: Figure S5C) expression of *cpl-1*. Meanwhile, knockdown of *cpl-1* also reduced fat accumulation in *daf-2* and *rict-1* mutants (Fig. 3a). However, in *tph-1* mutants, knockdown of *cpl-1* did not affect body fat storage (Fig. 3a). Further, we examined the expressions of genes that are critically involved in the pathways. Knockdown of *cpl-1* did not affect the expressions of genes related to insulin signaling and TOR signaling pathways, but sharply increased the expression of serotonin signaling molecules, including *tph-1*, *mod-1*, *ser-1*, *ser-4*, and *ser-6* (Fig. 3b). Moreover, in N2 worms, knockdown of *cpl-1* significantly elevated the levels of serotonin (Fig. 3c). In *C. elegans*, besides body fat, serotonin also regulates various behaviors, such as feeding [19] and olfactory learning ability for avoiding pathogenic bacteria [20]. Since knockdown of *cpl-1* activated serotonin signaling, the serotonin-mediated behaviors should also be elevated. As expected, in N2 worms, knockdown of *cpl-1* increased their pumping rates (Fig. 3d) and olfactory learning abilities for avoiding pathogenic bacteria (Fig. 3e). Consistently, compared with N2 worms, three *cpl-1* mutant alleles *ok360*, *qx304*, and *yq89* also had higher levels of serotonin signaling molecules (Fig. 3f), serotonin content (Fig. 3g), pumping rate (Fig. 3h), and olfactory learning ability (Fig. 3i), although *cpl-1(qx304)* mutation did not affect the expressions of genes involved in insulin signaling and TOR signaling pathways (Additional file 10: Figure S6A). Together, the results suggest that serotonin signaling is required for the effects of CPL-1 inhibition on body fat loss in *C. elegans*.

### Knockdown of *cpl-1* in the intestine and hypodermis promoted serotonin synthesis in ADF neurons in *C. elegans*

In *C. elegans*, fat depots are primarily stored in the intestinal and skin-like epidermal/hypodermal cells [21]. As a widely distributed protein in worms, CPL-1 is also expressed in the intestine and hypodermis [16]. Here, we introduced an extrachromosomal array (yqEx688, *P*_*cpl-1*_*cpl-1::mChOint*) expressing CPL-1 fusion mChOint fluorescence protein into the RT258 (*unc-119(ed3)III;pwls50*) strain. The punctate of CPL-1::mChOint overlapped very well with the GFP-tagged lysosomal membrane protein LMP-1 (LMP-1::GFP) in the intestinal and hypodermal cells (Additional file 11: Figure S7), confirming the location of CPL-1 in lysosomes of the cells.

**Fig. 4:**
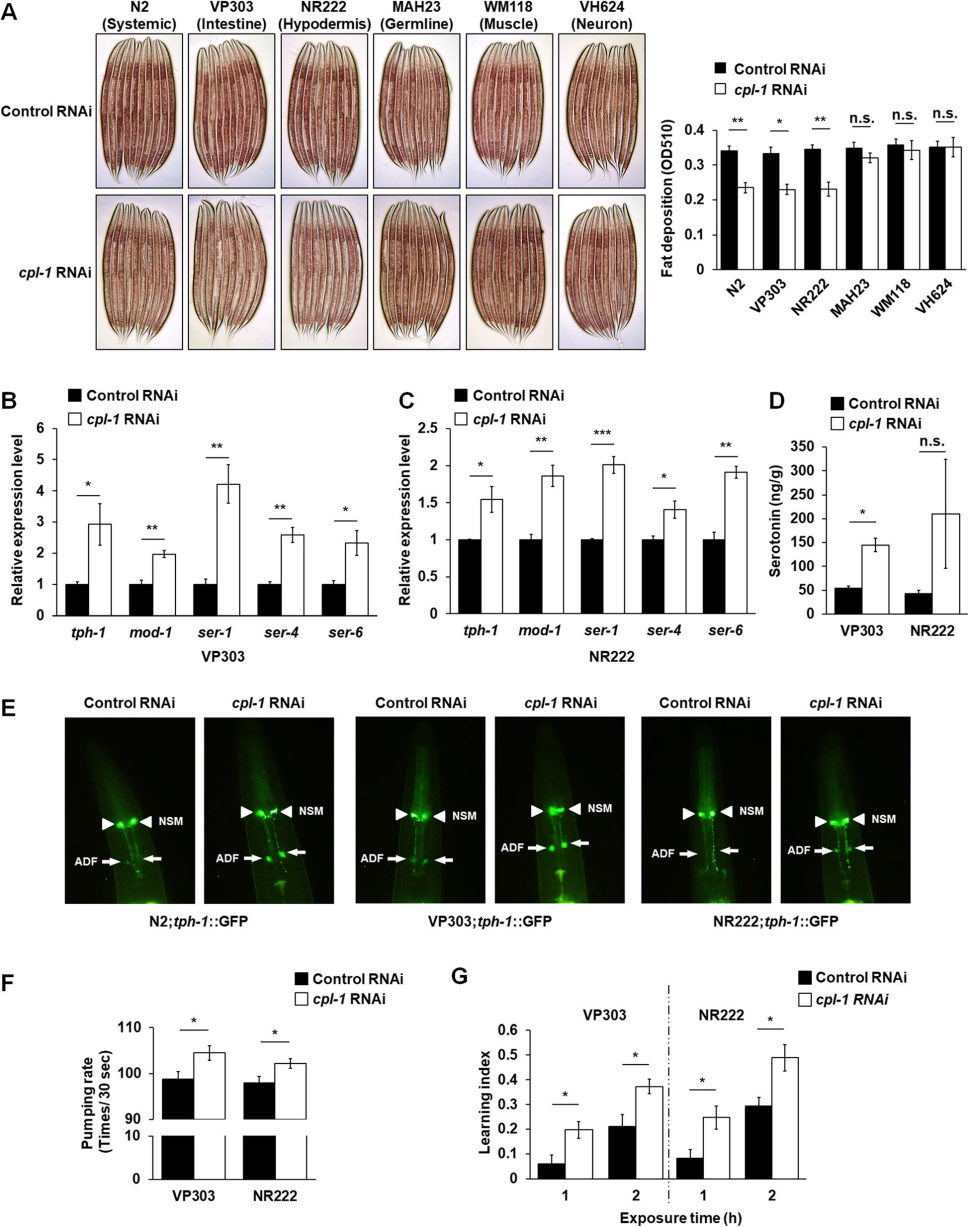
Knockdown of *cpl-1* in the intestine and hypodermis promoted serotonin synthesis in ADF neurons*.* **a** Representative images and quantification of Oil Red O staining in tissue-selective RNAi strains: intestine-restricted VP303 (*rde-1(ne219) V;kzls7*), hypodermis-restricted NR222 (*rde-1(ne219) V;kzls9*), germline-restricted MAH23 (*rrf-1(pk1417) I*), muscle-restricted WM118 (*rde-1(ne300) V;nels9 X*), and neurons-restricted VH624 (*rhIs13 V; nre-1(hd20) lin-15B(hd126) X*), during control RNAi or *cpl-1* RNAi treatment. The data were obtained from 3 independent experiments, and 3000 stained worms were used in each experiment. **b**, **c** Real-time PCR analysis of genes involved in the serotonin signaling pathway in intestine-selective *cpl-1* knockdown worms (**b**) and hypodermis-selective *cpl-1* knockdown worms (**c**). *act-1* was used as the reference gene in real-time PCR analysis, *n* = 3 independent growths. **d** The serotonin levels in intestine-selective (VP303) and hypodermis-selective (NR222) *cpl-1* knockdown worms; the data were based on 3 independent experiments. **e** The expression of *tph-1::gfp* in systemic, intestine-selective (VP303), and hypodermis-selective (NR222) *cpl-1* knockdown worms; *tph-1* expression was visible in NSM (arrowheads) and in ADF (arrows). For GFP fluorescence quantification, the data were obtained from 3 independent experiments and 30 worms were imaged and qualified with the level of fluorescence intensity. **f** The pharyngeal pumping rate in intestine-selective (VP303) and hypodermis-selective (NR222) *cpl-1* knockdown worms; the data were obtained from 3 independent experiments, and 30 worms were used in each experiment. **g** The two-choice olfactory preference assays to PA14 and HT115 bacteria in intestine-selective (VP303) and hypodermis-selective (NR222) *cpl-1* knockdown worms, *n* = 3 independent growths. All data in **a**–**d**, **f**, and **g** are presented as mean ± SEM. Two-tailed Student’s *t* test was used for statistical analysis. *p < 0.05; **p < 0.01; ***p < 0.001. n.s., not significant

Given that systemic knockdown of *cpl-1* reduced fat accumulation in *C. elegans* through activating serotonin signaling, we asked whether local knockdown of *cpl-1* in fat storage tissues could also lead to similar results. To answer the question, we utilized several strains whose RNAi knockdown was valid only in specific tissues, including intestine-restricted VP303 (*rde-1(ne219) V;kzls7*), hypodermis-restricted NR222 (*rde-1(ne219) V;kzls9*), germline-restricted MAH23 (*rrf-1(pk1417) I*), muscle-restricted WM118 (*rde-1(ne300) V;nels9 X*), and neuron-restricted VH624 (*rhIs13 V; nre-1(hd20) lin-15B(hd126) X*), to perform tissue-selective knockdown of *cpl-1*. All *cpl-1* knockdown worms had similar developmental rates to their corresponding controls (Additional file 7: Table S4). Intriguingly, knockdown of *cpl-1* in the intestine and hypodermis, rather than in the germline, muscle, or neuron, remarkably reduced fat accumulation (Fig. 4a) and elevated the expression of the serotonin signaling molecules (Fig. 4b, c and Additional file 12: Figure S8A). Similar to the knockdown of *cpl-1* in the whole body (Fig. 3c), knockdown of *cpl-1* in the intestine and hypodermis also elevated worm serotonin levels (Fig. 4d). To confirm that the tissue-selective knockdown was effectively restricted in a corresponding tissue, we introduced yqEx688 (*P*_*cpl-1*_*cpl-1::mChOint*) into the tissue-selective knockdown strains and found that the *cpl-1* knockdown worms had significantly lower CPL-1::mChOint expressions in the specific tissues than their controls (Additional file 12: Figure S8B). Therefore, local knockdown of *cpl-1* in fat storage tissues, such as the intestine and hypodermis, suppresses fat accumulation and activate serotonin signaling.

As a classical neurotransmitter, serotonin is only produced from several neurons in *C. elegans*. In the neurons, including head sensory neuron ADF, pharyngeal neuron NSM, and hermaphrodite-specific neuron HSN, tryptophan hydroxylase TPH-1 as a critical rate-limiting enzyme is required for serotonin biosynthesis [11]. Thus, to assess the effects of CPL-1 inhibition in the intestine and hypodermis on central serotonin synthesis, we crossed the P*tph-1::gfp* line with N2, the VP303, or the NR222 strains and performed knockdown of *cpl-1* in the whole body, intestine, and hypodermis, respectively. Notably, an increased expression of TPH-1 was observed in ADF neurons in the *cpl-1* knockdown worms (Fig. 4e). In *C. elegans*, serotonin from ADF neurons and serotonin from NSM neurons specifically regulate distinct physiological functions. Unlike the specific role of serotonin from NSM neurons in foraging regulation [22, 23], serotonin from ADF neurons specifically regulates body fat loss, feeding, and olfactory learning ability for avoiding pathogenic bacteria [20, 24, 25]. Consistent with this notion, we found that mutation of *cpl-1* and knockdown of *cpl-1* in the whole body, intestine, and hypodermis not only induced body fat loss (Figs. 2b, d and 4a) but also elevated worm pumping rates (Figs. 3d, h and 4f) and olfactory learning abilities for avoiding pathogenic bacteria (Figs. 3e, i and 4g). The results suggest that knockdown of *cpl-1* in the intestine and hypodermis resembles systemic CPL-1 inactivation and induces body fat loss in *C. elegans* by promoting serotonin synthesis in ADF neurons.

### Knockdown of *cpl-1* induced body fat loss via a central serotonin signaling

**Fig. 5:**
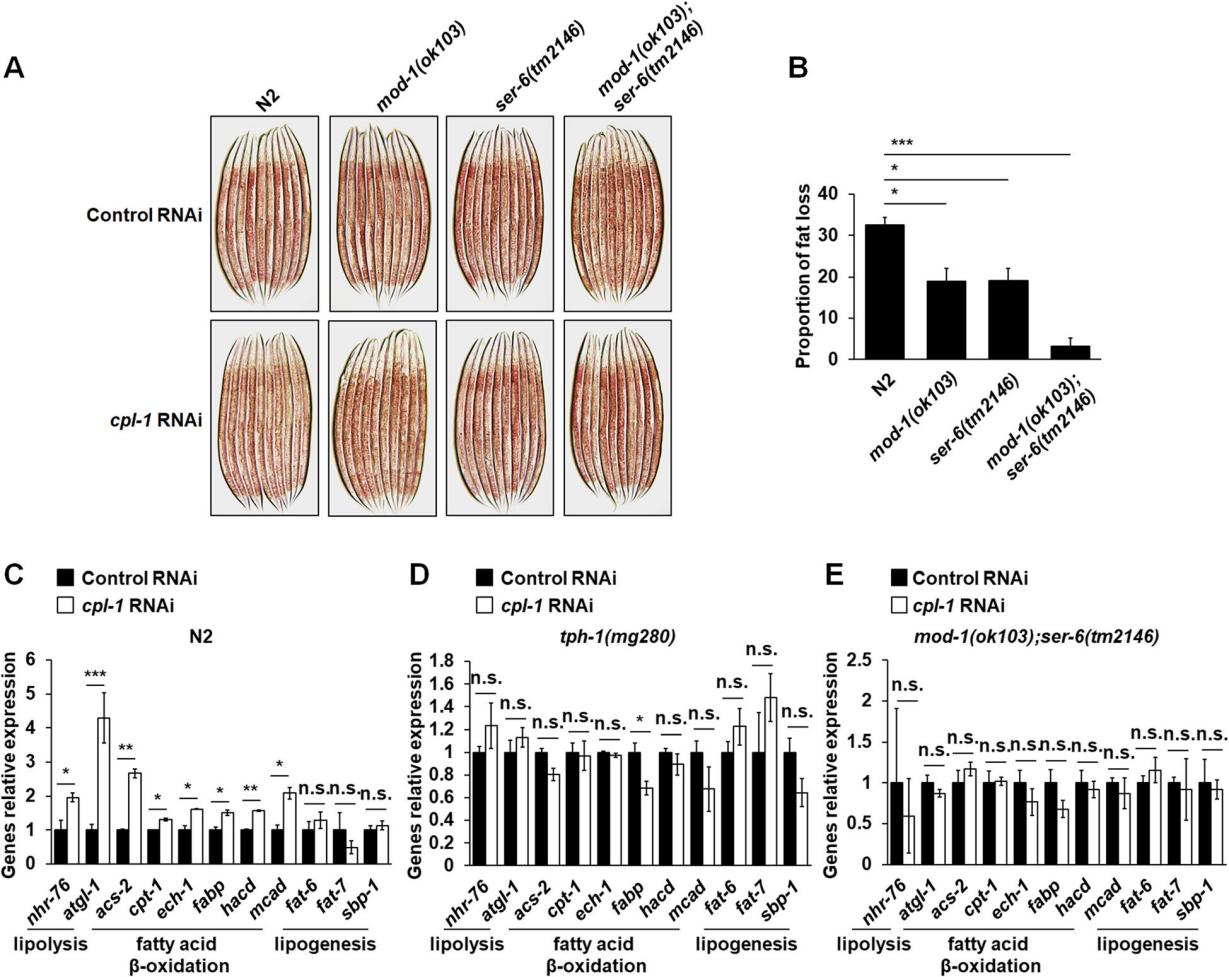
Knockdown of *cpl-1* induced body fat loss via a central serotonin signaling. **a** Representative images of Oil Red O staining in N2, *mod-1(ok103)*, *ser-6(tm2146)*, and *mod-1(ok103);ser-6(tm2146)* worms fed with control or *cpl-1* RNAi bacteria. **b** Quantification of Oil Red O staining in N2, *mod-1(ok103)*, *ser-6(tm2146)*, and *mod-1(ok103);ser-6(tm2146)* worms fed with control or *cpl-1* RNAi bacteria; the data were presented as the proportion of fat loss, *n* = 3 independent growths. **c**–**e** Real-time PCR analysis of genes involved in lipolysis, fatty acid β-oxidation, and lipogenesis in N2 (**c**), *tph-1(mg280)* (**d**), and *mod-1(ok103);ser-6(tm2146)* (**e**) worms fed with control or *cpl-1* RNAi bacteria. *act-1* was used as the reference gene in real-time PCR analysis, *n* = 3 independent growths. The data in **b**–**e** are presented as mean ± SEM. For **b**, one-way ANOVA; for **c**–**e**, two-tailed Student’s *t* test was used for statistical analysis. *p < 0.05; **p < 0.01; ***p < 0.001. n.s., not significant

In *C. elegans*, it has been well defined that a central serotonin signaling controls body fat loss. The central serotonin signaling, including octopaminergic G protein-coupled receptor SER-6 in AWB sensory neurons and serotoninergic chloride channel MOD-1 in URX body cavity neurons, integrates octopamine signaling with serotonin production in ADF neurons and hence induces lipolysis and fatty acid β-oxidation in the intestine [19, 24, 26]. Given that knockdown of *cpl-1* elevated serotonin synthesis in ADF neurons and expressions of *mod-1* and *ser-6* (Figs. 3 and 4), MOD-1 and SER-6 should also be required for the effects of CPL-1 inhibition on body fat loss. Consistent with the notion, although knockdown of *cpl-1* (Additional file 9: Figure S5B, S5C and Fig. 2c) still reduced fat deposition in either *mod-1(ok103)* or *ser-6(tm2146)* mutants, the extents of fat loss in the mutants were significantly lower than those in N2 worms (Fig. 5a, b). And *mod-1(ok103);ser-6(tm2146)* double mutation could entirely abolish CPL-1 inhibition-mediated fat loss (Fig. 5a, b). Meanwhile, knockdown of *cpl-1* did not affect the developmental rates of the mutant strains (Additional file 7: Table S4). Further, we examined the expressions of genes that are responsible for lipogenesis, lipolysis, and fatty acid β-oxidation. Knockdown of *cpl-1* significantly increased the expressions of genes involved in lipolysis and fatty acid β-oxidation (Fig. 5c). Similarly, the expressions of genes related to lipolysis and fatty acid β-oxidation were also increased in *cpl-1* mutant *qx304* (Additional file 13: Figure S9A). However, in *tph-1* (Fig. 5d) and *mod-1;ser-6* (Fig. 5e) mutants, the expressions of the genes were not almost affected by the knockdown of *cpl-1*. The results suggest that knockdown of *cpl-1* activates lipolysis and fatty acid β-oxidation in *C. elegans* via a central serotonin signaling involving MOD-1 and SER-6. Thus, a previously undefined circuit that peripheral CPL-1 inhibition promotes central serotonin signaling to regulate body fat loss is identified in *C. elegans*.

### Cathepsin L knockout promoted fat loss and central serotonin synthesis in HFD-fed mice

**Fig. 6:**
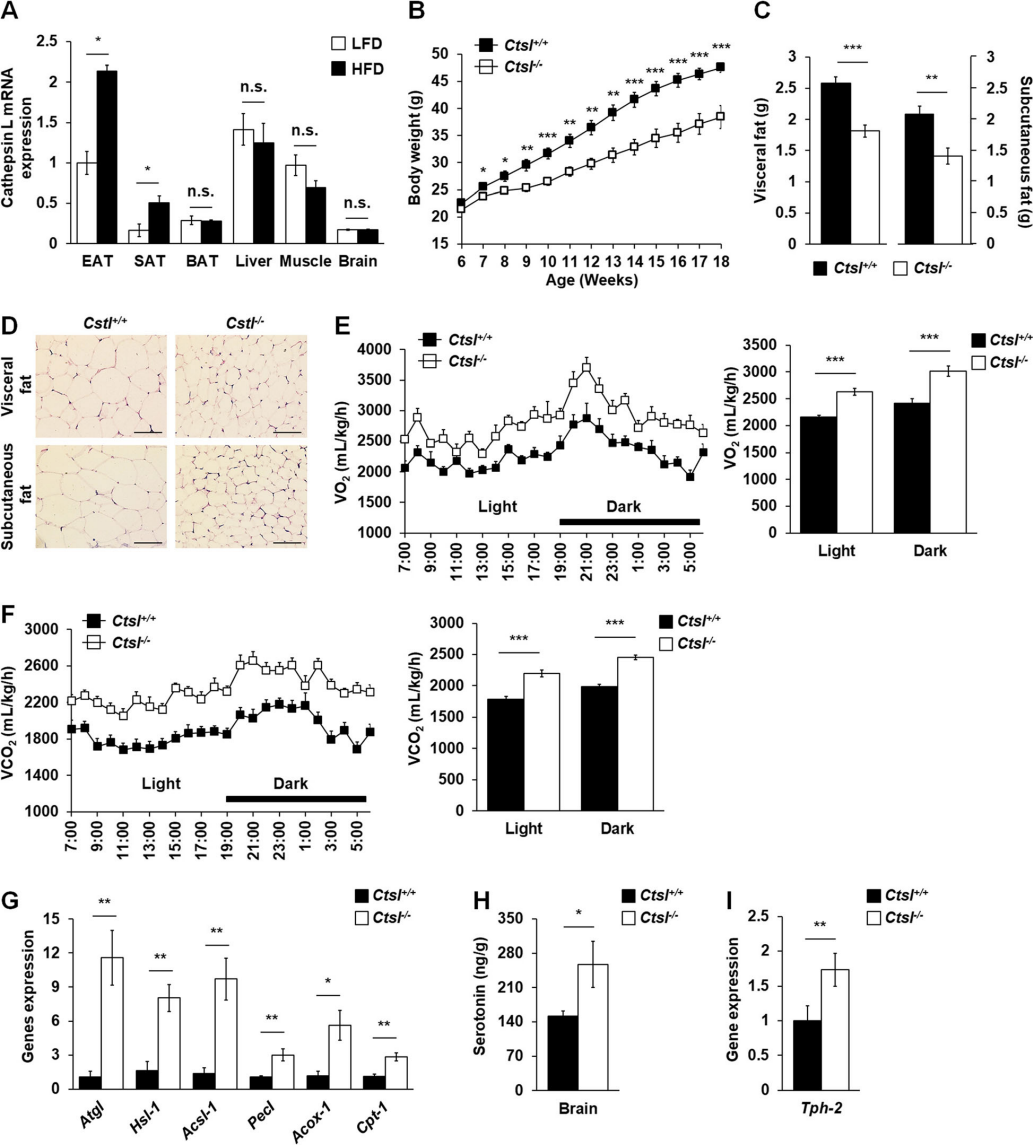
Cathepsin L knockout promoted fat loss and central serotonin synthesis in HFD-fed mice. **a** Real-time PCR analysis of cathepsin L expression in metabolic and neuronal tissues in LFD- and HFD-fed mice. EAT, epididymal adipose tissue; SAT, subcutaneous adipose tissue; BAT, brown adipose tissue. **b**–**i** Male 6-week-old *Ctsl*^*+/+*^ and *Ctsl*^*−/−*^ mice were fed with HFD for 12 weeks. **b** Body weight was recorded every week. **c** White adipose tissue weights were measured at 18 weeks old. **d** Hematoxylin-eosin staining in the epididymal fat and subcutaneous fat, scale bars 100 μm in length (× 200). **e**, **f** At 18 weeks old, the mouse metabolic parameters were measured during a 12-h light and 12-h dark cycle and the average for each group in the light or dark cycle. **e** Oxygen consumption (VO_2_) and **f** carbon dioxide production (VCO_2_). **g** Real-time PCR analysis of genes involved in lipolysis and β-oxidation in WAT. **h** The contents of serotonin in mice brain. **i** Real-time PCR analysis of the expression of *Tph-2* in mouse brain. *β-Actin* was used as the reference gene in real-time PCR analysis. The data in **a**–**c** and **e**–**i** are presented as mean ± SEM, *n* = 10 per group. *p < 0.05; **p < 0.01; ***p < 0.001. n.s. not significant in a nonparametric Mann-Whitney test

In *C. elegans*, the identification of the circuit, which peripheral CPL-1 inhibition promotes central serotonin synthesis to induce body fat loss, originates from our initial observation that the expression of lysosomal cathepsin L-like protease CPL-1 elevated along with nutrient-induced fat accumulation. Thus, to explore whether there is a similar circuit to regulating fat storage in the mammal system, we should firstly demonstrate whether there is increased cathepsin L expression in some fat storage tissues among with diet-induced obesity. Therefore, 6-week-old wild-type mice were fed with HFD and low-fat diet (LFD). After 12 weeks, cathepsin L expressions were detected in metabolic and neuronal tissues, such as adipose tissue, liver, muscle, and brain in mice. Cathepsin L was widely distributed in all tested tissues. However, only in WAT including epididymal and subcutaneous adipose tissue cathepsin L expressions in obese HFD-fed mice were higher than those in lean LFD-fed mice (Fig. 6a), suggesting cathepsin L may be required for WAT fat accumulation. Therefore, to test this hypothesis, cathepsin L-deficient (*Cstl*^*−/−*^) mice and their littermate control (*Cstl*^*+/+*^) mice were fed with LFD or HFD for 12 weeks. In LFD-fed mice, although there were no differences in body weight between *Ctsl*^*−/−*^ mice and control *Ctsl*^+/+^ mice (Additional file 14: Figure S10A), *Ctsl*^*−/−*^ mice had lower subcutaneous fat weight (Additional file 14: Figure S10B), higher food intake (Additional file 14: Figure S10C), and more O_2_ consumption and CO_2_ production (Additional file 14: Figure S10D and S10E) than *Ctsl*^+/+^ mice. Moreover, compared with those in *Ctsl*^*+/+*^ mice, brain serotonin levels were also increased in *Ctsl*^*−/−*^ mice, although the increase did not reach statistical significance between the two groups (Additional file 14: Figure S10F).

Like LFD-fed *Ctsl*^*−/−*^ mice, HFD-fed *Ctsl*^*−/−*^ mice also had a higher level of food intake than HFD-fed *Ctsl*^+/+^ mice (Additional file 14: Figure S10C). However, the HFD-fed *Ctsl*^*−/−*^ mice had a lower body (Fig. 6b) and WAT weight gain and fat accumulation (Fig. 6c, d) than HFD-fed *Ctsl*^+/+^ mice. Further, we detected the energy expenditure in mice. In the HFD-fed mice, despite there were no differences in O_2_ consumption and CO_2_ production between *Ctsl*^*−/−*^ and *Ctsl*^+/+^ mice when the metabolic data were normalized per animal (Additional file 15: Figure S11 A and S11B), *Ctsl*^*−/−*^ mice had a significantly higher metabolic rates than *Ctsl*^+/+^ mice when the data were normalized to body weight (Fig. 6e, f). Correspondingly, the expressions of genes responsible for lipolysis and fatty acid β-oxidation (Fig. 6g) were enhanced in WAT in HFD-fed *Ctsl*^*−/−*^ mice. Notably, compared with HFD-fed *Ctsl*^*+/+*^ mice, HFD-fed *Ctsl*^*−/−*^ mice had higher brain serotonin levels (Fig. 6h) and central tryptophan hydroxylase *Tph2* expression (Fig. 6i). Together, the results indicate that a genetic deficiency of cathepsin L enhances brain serotonin production and promotes fat loss in HFD-fed mice, suggesting a circuit including peripheral cathepsin L inhibition and central serotonin synthesis may also induce fat loss in HFD-fed mice.

### Peripheral inhibition of cathepsin L induced fat loss in HFD-fed mice through promoting central serotonin synthesis

**Fig. 7:**
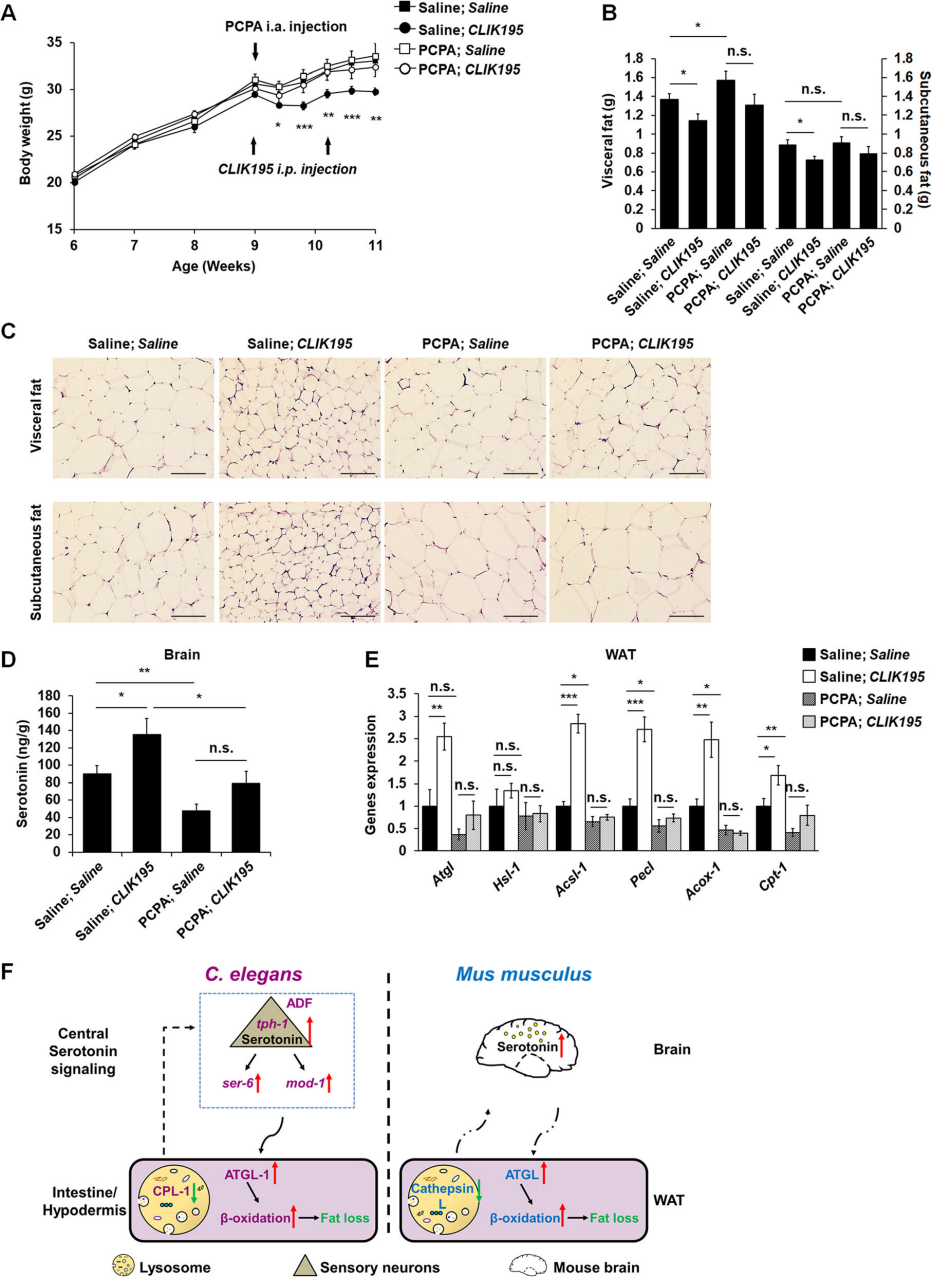
Peripheral cathepsin L inhibition induced fat loss in HFD-fed mice through promoting central serotonin synthesis. **a**–**e** Male 6-week-old wild-type mice were fed with HFD for 5 weeks. At 9 weeks old, 30 mg kg^−1^ of PCPA was injected intracranially into the ventricles; 100 mg kg^−1^ of CLIK195 was intraperitoneally injected at 9 and 10 weeks old. **a** Body weight of the mice was measured every 3 days. **b** White adipose tissue weights were measured at 11 weeks old. **c** Hematoxylin-eosin staining in the epididymal fat and subcutaneous fat, scale bars 100 μm in length (× 200). **d** The levels of serotonin in the brain. **e** Real-time PCR analysis of genes involved in lipolysis and β-oxidation in WAT. *β-Actin* was used as the reference gene in real-time PCR analysis. **f** In both *C. elegans* and mice, peripheral cathepsin L inhibition lowers fat accumulation through promoting central serotonin synthesis. In *C. elegans* (left), CPL-1 inhibition in the intestine and hypodermis promotes serotonin synthesis in ADF neurons. Thus, a central serotonin signaling, involving octopaminergic SER-6 and serotonin from ADF neurons and serotonergic MOD-1, is activated and hence stimulates lipolysis and β-oxidation in the intestine and hypodermis, which results in fat loss. In HFD-fed mice (right), peripheral cathepsin L inhibition activates lipolysis and β-oxidation and induces fat loss in WAT through promoting central serotonin production. The data in **a**, **b**, **d**, and **e** are presented as mean ± SEM, *n* = 10 per group. *p < 0.05; **p < 0.01; ***p < 0.001. n.s. not significant in a nonparametric Mann-Whitney test

Given that a similar phenotype between *cpl-1*-deficient worms and HFD-fed *Ctsl*^*−/−*^ mice, we asked whether peripheral inhibition of cathepsin L activity could induce WAT fat loss through promoting central serotonin synthesis. Thus, we fed wild-type mice with HFD for 3 weeks and then grouped the mice into four treatment groups: group 1, received intracranial (i.a.) and intraperitoneal (i.p.) injection of saline as control; group 2, received i.a. injection of saline and i.p. injection of cathepsin L inhibitor CLIK195; group 3, received i.a. injection of tryptophan hydroxylase inhibitor p-hlorophenylalanine (PCPA) and i.p. injection of saline; and group 4, received i.a. injection of PCPA and i.p. injection of CLIK195. Peripheral CLIK195 administration (group 2) reduced body weight gain (Fig. 7a) and adipose accumulation (Fig. 7b, c) obviously in HFD-fed mice. However, combined central PCPA and peripheral CLIK195 administration (group 4) entirely abolished the effects of CLIK195 on body weight gain (Fig. 7a) and adipose accumulation (Fig. 7b, c). Meanwhile, compared with the control mice (group 1), the peripheral CLIK195-treated mice (group 2) had higher brain serotonin levels (Fig. 7d). By contrast, central PCPA treatment significantly reduced brain serotonin levels (group 3) and resisted the effects of CLIK195 treatment on brain serotonin levels (group 4) (Fig. 7d). Along with higher brain serotonin levels, the peripheral CLIK195-treated mice (group 2) also had higher levels of WAT lipolysis and fatty acid β-oxidation than the other three groups. And PCPA treatment (group 4) also abolished the action of CLIK195 treatment on WAT lipolysis and fatty acid β-oxidation (Fig. 7e). Therefore, a conserved circuit that peripheral inhibition of cathepsin L activity promotes central serotonin synthesis also controls fat accumulation in HFD-fed mice.

## Discussion

In mammals, adipose tissue is a primary fat storage organ. WAT lipometabolic imbalance, especially excess fat accumulation, is closely related to the development of obesity and associated insulin resistance, diabetes, and cardiovascular disease [27–29]. Thus, it is crucial to understand the regulation of fat storage or loss. In the present study, we select a classic model animal *C. elegans* to establish a model of fat accumulation. Further, utilizing to the model, we demonstrate an increased expression of lysosomal cathepsin L-like protease CPL-1 along with nutrient-induced fat accumulation and hence reveal a previously undescribed molecular circuit that CPL-1 inhibition in fat storage tissues promotes serotonin production in neurons and, in turn, induces body fat loss in *C. elegans* (Fig. 7f, left). Finally, a similar circuit is also recapitulated in HFD-fed mice (Fig. 7f, right).

Long-term excess energy intake, for example, an increase in dietary saturated fats and sugars, fundamentally results in the development of obesity and comorbidity metabolic diseases in mammals. Glucose is an essential nutrient and the main component of metabolism and energy production. In *C. elegans*, like its action in mammals, dietary glucose supplementation increases fat accumulation [30, 31] and hence leads to some pathophysiological changes, such as accelerated aging and life shorting [32, 33]. Palmitic acid is one of the most common saturated fatty acids in the human body [34] and the primary saturated fatty acid in *E. coli* OP50 membrane [35]. Therefore, in this study, to establish a *C. elegans* model of nutrient-induced fat accumulation, glucose and palmitic acid are supplemented into the NGM plates.

Across all metazoans, fat accumulation is conserved controlled by a series of critical enzymes that catalyze multistep conversions from acetyl CoA to TAG [18]. In *C. elegans*, some key rate-limiting enzymes, such as acetyl CoA carboxylase and fatty acid synthase, are expressed at all developmental stages, and their deficiency can result in early larval arrest, suggesting fat accumulation may be an essential requirement for larval and adult normal growth [18]. On the other hand, the formation of dauer, which is a phenotype of post-embryonic developmental arrest in *C. elegans*, is characterized by increased fat accumulation and altered metabolism [11]. Thus, in *C. elegans*, fat accumulation and post-embryonic development rate may influence each other. To avoid the influence, we must develop a *C. elegans* model with increased fat deposition and without altered developmental rate. In the present study, as shown in Fig. 1a, a short-term nutrient supplement from L4 to adult is performed. Utilizing such a short-term supplement, both glucose and palmitic acid induce fat accumulation and increase *cpl-1* expression in N2 worms, yet they do not affect worm developmental rate (Fig. 1, Additional file 5: Figure S3, and Additional file 2: Table S1).

As a proteolytic enzyme localized in lysosomes, CPL-1 has been implicated in embryogenesis, development, and phagosomal degradation of apoptotic germ cells in *C. elegans* [16, 17]. In the previous studies, to enucleate the relative role of CPL-1 in worm development and embryogenesis, several *cpl-1* mutants have been utilized. The embryos of *cpl-1* mutant *ok360* are lethal, suggesting that CPL-1 is essential for the degradation of yolk proteins during worm embryogenesis [17]. However, high embryonic lethality does not mean abnormal post-embryonic development. Due to maternal providence of CPL-1, the heterozygous *cpl-1(ok360)* F1 progeny usually develops to adults without visible defects in growth rate, molting, and brood size [16, 17]. Moreover, two weak loss-of-function mutants of *cpl-1*, *qx304*, and *yq89* also develop normally when cultured at 20 °C [16]. Consistent with the previous observations, all tested worms in this study, including *qx304* and *yq89* mutants, homozygous *cpl-1(ok360)* F1 progenies generated by heterozygous mothers and *cpl-1* knockdown worms, develop normally when cultured at 20 °C (Additional file 6: Table S3 and Additional file 7: Table S4). Utilizing the normally developed *cpl-1*-deficient and knockdown worms, we demonstrate that CPL-1 inhibition reduces basal and nutrient-induced fat accumulation (Fig. 2 and Additional file 8: Figure S4), although its role in regulating nutrient-induced fat accumulation is yet to be thoroughly studied in *C. elegans*.

Some conserved signaling pathways across metazoans, such as insulin, TOR, and serotonin pathways, have been reported to regulate body fat in *C. elegans* [11, 18]. As the most well-studied metabolic regulating pathway, insulin signaling connects nutrient levels to many cellular processes, such as fat metabolism, stress response, and longevity [36]. In *daf-2(e1370)* mutants, the deficiency of insulin receptor-like gene increases body fat content [37, 38]. As a phosphatidylinositol kinase-related family member, TOR regulates cell growth and proliferation in response to nutrient levels [11]. In *C. elegans*, *rict-1* encodes a component of TOR complex, and the deficiency of *rict-1(ft7)* increases body fat [39]. As a neuromodulator, serotonin controls various food-related behaviors and physiological processes in *C. elegans* [11, 18, 23]. In *tph-1(mg280)* mutants, the failure to serotonin synthesis elevates fat storage [24, 38, 40]. In contrast, exogenous serotonin addition into the NGM plates reduces TAG levels in N2 worms [19, 24, 26]. In this study, similar to those in N2 worms, knockdown of *cpl-1* reduces body fat content in *daf-2* and *rict-1* mutants. In contrast, deficiency of *tph-1* blocks the action of *cpl-1* knockdown. Further, utilizing *cpl-1*-deficient and knockdown worms, we find that CPL-1 inhibition elevates worm serotonin signaling, suggesting serotonin signaling is required for CPL-1 inhibition-mediated fat loss.

In *C. elegans*, serotonin is only synthesized in a few central neurons, such as ADF and NSM neurons [41, 42]. Serotonin from ADF neurons specifically mediates body fat loss [43]. Recently, concerning the serotonin-mediated fat loss, a central serotonin signaling, which integrates octopamine signaling with serotonin production, has been deciphered in *C. elegans* [24, 26]. In the central serotonin circuit, exogenous octopamine treatment and octopamine released from RIC neurons regulate serotonin synthesis in ADF neurons via octopaminergic receptor SER-6 in AWB neurons. In turn, serotonin from ADF neurons promotes the secretion of Phe-Met-Arg-Phe-NH2 (FMRFamide)-like neuroendocrine peptide FLP-7 via serotoninergic receptor MOD-1 in URX neurons. Further, FLP-7 stimulates lipolysis and fatty acid β-oxidation and hence leads to fat loss in the intestine via the NPR-22/NK2R receptor [24, 26]. The central serotonin circuit has well underlain serotonin-mediated fat loss in *C. elegans*, but it prompts us to test whether the peripheral signaling could be transmitted to initiate the central serotonin signaling. In this study, we demonstrate that CPL-1 inhibition in peripheral fat storage tissues is such an initiator. In the context of *cpl-1* knockdown, we recapitulate the central serotonin signaling and demonstrate that peripheral CPL-1 inhibition indeed promotes central serotonin signaling to induce fat loss (Figs. 4 and 5), although a gap between peripheral CPL-1 inhibition and central serotonin synthesis is yet to be thoroughly studied in *C. elegans*.

In mammals, owing to the separation of the blood-brain barrier, there are two self-governed compartments of serotonin production and action. There are two rate-limiting enzymes that tryptophan hydroxylase Tph1 and Tph2 control peripheral and central serotonin synthesis, respectively [44]. Although 95% serotonin in the body is produced from peripheral tissues, central serotonin substantially regulates mammalian energy balance [45]. Central serotonin abatement, such as pharmacological inhibition of central serotonin synthesis in rats and induced necrosis of Pet-1^+^ serotonin neurons in mice, weakens thermogenesis and elevates fat accumulation in adipose tissue [46, 47]. Thus, like serotonin from ADF neurons in *C. elegans*, murine central serotonin also negatively regulates body fat accumulation. Supporting the notion, in this study, the lean HFD-fed *Ctsl*^*−/−*^ mice has higher levels of brain serotonin and WAT lipolysis and fatty acid β-oxidation than the obese HFD-fed *Ctsl*^+/+^ mice (Fig. 6). In HFD-fed wild-type mice, peripheral administration of cathepsin L inhibitor CLIK195 reduces body weight gain and WAT adipogenesis and elevates brain serotonin levels and WAT lipolysis and fatty acid β-oxidation, while the intracranial injection of tryptophan hydroxylase inhibitor PCPA abrogates the effects of peripheral CLIK195 infusion (Fig. 7). Therefore, similar to that in *C. elegans*, a circuit, which peripheral cathepsin L inhibition promotes central serotonin synthesis, also induces fat loss in HFD-fed mice.

## Conclusions

In conclusion, this study reveals a previously undescribed molecular mechanism that peripheral CPL-1/cathepsin L inhibition induces fat loss in *C. elegans* and mice through promoting central serotonin signaling (Fig. 7f). As potential therapeutic targets of obesity and associated metabolic disorders, the main modulators of the novel circuit merit combined evaluating in the future experimental animals and humans.

## Methods

### *C. elegans*

Worms were maintained according to the standard protocols [48] unless specifically noted. N2 Bristol was used as a wild-type reference strain, and the following mutant strains were obtained from *Caenorhabditis* Genetics Center (CGC): VC322 (*+/nT1 IV;cpl-1(ok360)/nT1 V*), *daf-2(e1370) III*, *rict-1(ft7) II*, *tph-1(mg280) II*, VP303 (*rde-1(ne219) V;kzls7*), NR222 (*rde-1(ne219) V;kzls9*), MAH23 (*rrf-1(pk1417) I*), WM118 (*rde-1(ne300) V;nels9 X*), VH624 (*rhIs13 V; nre-1(hd20) lin-15B(hd126) X*), RT258 (*unc-119(ed3)III;pwls50*), GR1366 *mgls42[tph-1::GFP+pRF4(rol-6(su1006))]*, *mod-1(ok103) V*, and *ser-6(tm2146)IV*. The *cpl-1* mutants *qx304* and *yq89* and CPL-1::mChOint transgenic line yqEx688 (*P*_*cpl-1*_*cpl-1::mChOint*) were as gifts from Professor Yang, Chonglin (the Institute of Genetics and Developmental Biology, Chinese Academy of Sciences). The worms expressing lipid droplet marker DHS-3::GFP (LIU1 (*ldrIs1 [dhs-3p::dhs-3::GFP + unc-76(+)]*)) were gifts from Professor Liu, Pingsheng (State Key Laboratory of Biomacromolecules, Institute of Biophysics, Chinese Academy of Sciences). All worms were synchronized for experiments by hypochlorite, hatched in M9 buffer (3 g KH_2_PO_4_, 6 g Na_2_HPO_4_, 5 g NaCl, 1 mL 1 M MgSO_4_, added H_2_O to 1 l) to the first larval (L1) stage, then seeded on plates.

To develop the nutrient-induced fat accumulation, we added glucose (at 1 mM and 5 mM) or palmitic acid (at 0.02 mM and 0.2 mM) into the NGM. The glucose was added into the sterile NGM after filtration sterilization. The palmitic acid was directly added into the NGM and sterilized by dry heat sterilization. For nutrient supplementation treatment, worms were added to the nutrient-supplemented plates since the L4 stage. After that, the worms were cultured for 16 h and harvested at adult.

### Mice

C57BL/6 WT mice were purchased from Vital River Laboratory Animal Technology Co. Ltd. (Beijing, China). Cathepsin L-deficient *Cstl*^*−/−*^ mice and their littermate control *Cstl*^*+/+*^ mice (males, C57BL/6/S129 background) were produced by *Ctsl*^*+/−*^ heterozygous breeding pairs as the previous study [2]. All research protocols were conducted with the approval of the Hefei University of Technology Standing Committee on Animals. Mice housed in a pathogen-free facility at 22 ± 2 °C with a 12/12-h light/dark cycle were given irradiated food and autoclaved water.

To induce obesity, male 6-week-old *Cstl*^*−/−*^ and *Cstl*^*+/+*^ littermates were fed with HFD (D12451 diet containing 4.73 kcal/g and 45% kcal% fat; *n* = 10) and LFD (D12450B diet containing 3.85 kcal/g and 10% kcal% fat; *n* = 10) for 12 weeks. Their body weights were weekly measured. After 12 weeks, their energy expenditures were measured by a combined indirect calorimetry system (TSE Systems GmbH, Bad Homburg, Germany) for 2 days. Either oxygen consumption (VO_2_) or carbon dioxide production (VCO_2_) was continuously monitored during the next 24 h. At the end of the experiments, the mice were euthanized by CO_2_. All the tissues were rapidly isolated and weighed on ice, snap-frozen in liquid nitrogen, and stored at − 80 °C. Hematoxylin-eosin staining was performed in the epididymal fat and subcutaneous fat to show adipocyte size.

In order to examine the effects of peripheral cathepsin L inhibition on cerebral serotonin synthesis, 6-week-old male WT mice were fed with HFD for 5 weeks. At 9 weeks old, to inhibit cerebral serotonin synthesis, some of them were i.a. injected 30 mg kg^−1^ tryptophan hydroxylase inhibitor PCPA. To suppress the activities of peripheral cathepsin L, some of them were i.p. injected 100 mg kg^−1^ cathepsin L inhibitor CLIK195 at 9 and 10 weeks old. Since 9 weeks old, body weights of the mice were measured every 3 days. At 11 weeks old, the mice were euthanized by CO_2_, and their brains and adipose tissues were harvested.

### Glucose and palmitic acid content in *E. coli* and *C. elegans*

The glucose quantification was performed as described [49] with minor modifications. Briefly, bacteria were seeded on control NGM plates and glucose-supplementing NGM plates for 1 day. And then, bacteria were pelleted and lysed in water by ultrasonic decomposition and centrifuged to remove the sediment. As to the analysis in *C. elegans*, approximately 20,000 young adult nematodes grown on control NGM plates and glucose-supplementing NGM were harvested, washed, and aliquots were removed for protein determination. The remaining nematodes were lysed in water and centrifuged to remove the sediment. The aqueous extracts of *E. coli* or *C. elegans* were measured by a blood glucose meter (Omnitest Plus, B.BRAUN, Germany). For the palmitic acid assay, bacteria seeded on control NGM plates and NGM plates with palmitic acid for 1 day were pelleted, and C15:0 standard was added to the pellet. The mixture was subjected to simultaneous extraction and transmethylation by incubating for 1 h at 70 °C in 1 mL of 2.5% H_2_SO_4_ in methanol. Then, palmitic acid was analyzed by gas chromatography as previously described [50]. As to the analysis of palmitic acid in *C. elegans*, approximately 20,000 young adult nematodes grown on control NGM plates and NGM plates with 0.02 mM or 0.2 mM palmitic acid were harvested and washed. Palmitic acid in *C. elegans* was extracted with chloroform to methanol (1:1) and analyzed by gas chromatography as previously described [51]. For the assay of glucose and palmitic acid, four independent growth experiments were performed, and each assay was repeated in triplicate for each growth.

### Phenotypic analysis of the developmental rate

The developmental rate was observed as described [52] with minor modifications. Worms were grown on NGM plates with the OP50 diet at 20 °C. The eggs were collected by bleaching and washed three times with M9 buffer and allowed to hatch in M9 buffer for 18 h. After synchronization, the L1 larva was transferred to NGM plates and incubated at 20 °C. At 50 h (worms fed with OP50) or 60 h (worms fed with HT115) after synchronization, worms were washed off and mounted on agarose pads and examined on a compound microscope. The numbers of L4, adult, and gravid adult worms were visually counted based on the development of the vulva. For each condition, 3 independent experiments were performed, and at least 30 worms were scored in each test.

### Oil Red O staining and quantitation

The Oil Red O staining was performed as described [37] with minor modifications; 0.5% Oil Red O solution was prepared in 1,2-propanediol and allowed to rest on a bench for a couple of days. On the day of staining, the Oil Red O solution was filtered through a 0.22-μm syringe filter. To conduct Oil Red O staining, day 1 adult worms were washed from NGM plates with M9 buffer for three times. After gravitational settling in tubes, 1000 μL M9 buffer and 50 μL 10% paraformaldehyde were added and immediately frozen in − 80 °C. And then, these tubes were thawed and refrozen for three cycles. At the third time, tubes were allowed to thaw on ice for 30–40 min. After complete thawing, worms were washed three times in cold M9 buffer. Then, worms were dehydrated in 1,2-propanediol for 5 min and stained with 1 mL Oil Red O solution for overnight at room temperature. After staining, worms were washed in turn with 85% 1,2-propanediol and PBS and mounted on a 2% agarose slide for imaging.

For Oil Red O quantification, the stained worms were fully suspended in 1 mL M9 buffer with 0.1% Tween-20. And then, we pipetted 10-μL suspension on slides and counted the worm numbers in the suspensions. To obtain accurate worm numbers in each 10-μL suspension, we repeated independently 3 times and took the mean to eradicate any discrepancies. According to the mean, 3000 worms were fetched in an appropriate volume. After centrifugation, stained lipid from the worms was extracted with 200 μL ethanol and quantitated at OD 510 nm.

### Label-free quantitative analysis of lipid droplets using DHS-3::GFP worms

The label-free quantification of lipid droplets in DHS-3::GFP worms was performed as previously described [51]. All worms were anesthetized in a droplet of 100 mM sodium azide and mounted on fresh 2% agarose slides before imaging. To evaluate the size of lipid droplet, images of DHS-3::GFP worms were used to measure the diameter of the lipid droplets in the posterior of the intestine with the same area by Image-Pro Plus Version 6.0 (Media Cybernetics, USA). Thirty animals were measured for each condition, and three independent experiments were repeated.

### Thin-layer chromatography and measurement of triglycerides

Adult worms were washed from NGM plates with M9 buffer. After gravitational settling in tubes, the worms were incubated in 0.9% NaCl at room temperature for 20 min. And then, the worms were pelleted again by centrifugation and resuspended in 500 μL H_2_O. Using chloroform to acetone (1:1, v/v), lipids were extracted from 400-μL nematode suspension as described previously [19]. The solvent was removed by pure nitrogen gas, and the lipids were dissolved in chloroform and separated on TLC plates in hexane to diethyl ether to acetic acid (80:20:1, v/v) for 40 min. TAG was visualized by dipping the plates into a developing reagent (0.63 g MnCl_2_, 60 mL water, 60 mL methanol, and 4 mL concentrated sulfuric acid) for 6 s. And then, the TLC plates were briefly dried and heated at 100 °C for 30 min. Utilizing the 100-μL rest nematode suspension, we assessed total protein contraction with the BCA assay kit (Sangong). TAG was quantitated by densitometric scanning at 400 nm with triolein (Sigma) as a standard and presented as TAG mass pre-microgram protein.

### RNAi experiments

RNAi-mediated inactivation of *cpl-1* was performed as described [53]. The 1157-bp fragment of *cpl-1(T03E6.7)* was PCR amplified from worm cDNA with the forward primer (*XhoΙ*), 5′-GGCCTCGAGCCATTCAGCCAATACCGCAA, and the reverse primer (*XbaΙ*), 5′-CTCTCTAGAGCAAATAAAACTGACCCGTC. The PCR product was cloned into the T7 vector L4440 and transfected into HT115 bacteria. HT115 bacteria containing RNAi vector were cultured and added into the NGM plates. L1-stage wild-type or mutant worms were placed on the plates with RNAi bacteria and incubated at 20 °C to adulthood.

### Quantitative real-time PCR

Total RNA was isolated from worms or mouse tissues using TRIzol reagent (TaKaRa). cDNA was synthesized using PrimeScript™ RT Master Mix (TaKaRa) according to the manufacturer’s protocol. The cDNA was used as the template for real-time PCR (Bio-Rad MyiQ2 Real-time PCR System) in the presence of SYBR® Premix Ex Taq™ II (TaKaRa). All primer sequences for *C. elegans* and mouse experiments were listed in Additional file 16: Table S5 and Additional file 17: Table S6, respectively. Data were processed using the ΔΔCT method. *act-1* was used as the reference gene in *C. elegans*, and *β-Actin* was used as the reference gene in mice.

### Western blot analysis

Total proteins were extracted from worms using RIPA lysis buffer with total protease inhibitor (1 μM PMSF) and phosphatase inhibitor (Sangon). The protein concentration was assessed with the BCA assay kit (Sangong). Samples in equal amounts were separated on SDS-PAGE and transferred to PVDF membranes (Millipore). Immunoblot analysis was performed with the corresponding primary and secondary antibodies. Then, reactive bands were developed using the ECL kit (Thermo) in ImageQuant LAS4000 mini (GE Healthcare) and quantified using ImageQuant TL 7.0 software (GE Healthcare). The antibodies included the following: anti-β-actin antibody; rabbit monoclonal antibody (Sigma-Aldrich, SAB5500001, 1:1000); GFP (D5.1) XP® rabbit monoclonal antibody (Cell Signaling Technology, Inc., #2956S, 1:1000); goat anti-rabbit IgG (H+L secondary antibody (Boster Biological Technology Co. Ltd., BA1054, 1:5000); and rabbit anti-CPL-1 antibody made by ourselves (1:5000). As to the production of rabbit anti-CPL-1 antibody, *cpl-1* (NM_001269789) gene was amplified by PCR and constructed into the prokaryotic expression plasmid (pET28a). Then, the recombinant protein was expressed in the BL21 *E. coli* expression system and purified. After that, the recombinant protein was injected into the rabbit, and the serum was collected. Finally, we detected the titer at 1:256,000 using enzyme-linked immunosorbent assay and identified good specificity using western blot. The related data were listed in Additional file 18: Figure S12.

### Determination of serotonin levels

The levels of serotonin in *C. elegans* and the whole mouse brain were measured by UPLC-MS/MS as previously described [54] with minor modifications. Briefly, the tissues were precisely weighed and fully lysed with a 20-fold (w/v) volume of 2% formic acid in methanol (v/v). After centrifugation at 13,000*g* for 25 min at 4 °C, 500 μL of supernatants was spiked with 10 μL of isoproterenol hydrochloride. And then, the mixtures were extracted using 490 μL methanol by vortexing for 30 s. After centrifugation at 13,000*g* for 5 min at 4 °C, the supernatants were directly detected. Their concentrations were calculated by Xcalibur software based on the standard samples.

### Two-choice olfactory preference assays

The two-choice olfactory preference assays were performed as previously described [20] with minor modifications. Synchronized L1 worms were grown at room temperature. “Naive” animals were grown on standard NGM plates with OP50 or HT115. For training olfactory preference abilities of worms, 200 μL suspending pathogenic *Pseudomonas aeruginosa* PA14 was spread on one side of an NGM plate and 50 μL OP50 or HT115 suspension was used to make a small lawn on the other side of the plate. The training plates were incubated at 20 °C for 24 h before use. For the two-choice olfactory preference assays, OP50, HT115, and PA14 were resuspended at the same absorbance of 1.0 at 600 nm. And then, 25 μL PA14 were spotted onto one side of a plate and 25 μL OP50 or HT115 were spotted on the other side of the plate. The assay plates were air-dried for 5 h at room temperature. To perform two-choice olfactory preference assays, adult worms were firstly transformed to the training plates for 1 h or 2 h. And then, “trained” or “naive” worms were washed twice in S-basal buffer. One hundred to 300 worms were placed at the center of the assay plates and allow them to be equidistant from the 2 bacteria spots. After moving freely for 1 h, the numbers of worms on bacteria spots were counted.

### Statistical analysis

Data were presented as mean ± SEM. As indicated in the figure legends, Student’s *t* test, one-way ANOVA, or two-way ANOVA was used to evaluate the difference among various *C. elegans* assay groups. As to the mouse experiments, the comparisons between the two groups were assessed with the nonparametric Mann-Whitney test due to our small sample sizes and data abnormal distribution. GraphPad Prism 7.01 (GraphPad Software, Inc.) was used as the statistical software, and *p* < 0.05 was considered as statistically significant.

## Supplementary information

**Additional file 1: Figure S1.** The glucose and palmitic acid uptake by *E. coli* and *C. elegans*. (A) The concentration of glucose in *E. coli* seeded on NGM plates with the supplementation of 1 mM or 5 mM glucose. (B) The relative concentration of glucose in *C. elegans* induced by the supplementation of 1 mM or 5 mM glucose. (C) The relative proportion of palmitic acid content in *E. coli* seeded on NGM plates with the supplementation of 0.02 mM or 0.2 mM palmitic acid. (D) The relative proportion of palmitic acid content in *C. elegans* induced by the supplementation of 0.02 mM or 0.2 mM palmitic acid. The data were obtained from 4 growths. All data are presented as mean±SEM, ^**^p<0.01; ^***^p<0.001 and n.s. not significant by one-way ANOVA.

**Additional file 2: Table S1.** The effect of nutrient supplementation on developmental rate in N2 worms.

**Additional file 3: Figure S2.** The expression of GFP fluorescence in DHS-3::GFP worms induced by the supplementation of nutrients. (A) Immunoblot and quantification of GFP protein in DHS-3::GFP worms induced by the supplementation of glucose or palmitic acid. The band of GFP protein was counted as the quantification of GFP to ACT-1, *n*=3 independent growths. (B) Representative images of GFP fluorescence in DHS-3::GFP worms induced by the supplementation of glucose or palmitic acid. Scale bar represents 20 μm. (C) Distribution of the lipid droplet size (% lipid droplets), as measured from images of GFP fluorescence in DHS-3::GFP worms from (B). The data were obtained from 3 independent experiments and 30 worms were imaged and qualified with the level of fluorescence intensity. The data in (A) and (C) are presented as mean±SEM, ^**^p<0.01; ^***^p<0.001 and n.s. not significant by one-way ANOVA.

**Additional file 4: Table S2.** The change of cathepsin-like gene expression in *C. elegans* after supplementation of 5 mM glucose.

**Additional file 5: Figure S3.** The expression of cathepsin genes in *C. elegans* induced by nutrients supplementation. (A) Heatmap of cathepsin genes expression in N2 worms fed with 5 mM glucose at L4 stage for 16 h. See Additional file 4: Table S2 for the data. (B) Real-time PCR analysis of cathepsin genes in N2 worms induced by the supplementation of glucose or palmitic acid. *act-1* was used as reference gene in real-time PCR analysis, *n*=3 independent growths. The data in (B) are presented as mean±SEM, ^*^p<0.05; ^**^p<0.01 and n.s. not significant by one-way ANOVA.

**Additional file 6: Table S3.** The effect of *cpl-1* mutation on developmental rate in *C. elegans*.

**Additional file 7: Table S4.** The effect of *cpl-1* RNAi on developmental rate in multiple mutants.

**Additional file 8: Figure S4.** Functional inactivation of CPL-1 decreased the fat accumulation in *C. elegans.* (A) Representative images and quantification of DHS-3::GFP fluorescence in N2 and *cpl-1(qx304)* worms induced by the supplementation of glucose or palmitic acid. The data were obtained from 3 independent experiments and 30 worms were imaged and qualified. (B) Representative image of TLC and TAG contents in N2 and *cpl-1(qx304)* worms induced by the supplementation of glucose or palmitic acid, *n*=3 independent growths. (C) Representative images and quantification of DHS-3::GFP fluorescence in control or *cpl-1* knockdown worms induced by supplementation of glucose or palmitic acid. The data were obtained from 3 independent experiments and 30 worms were imaged and qualified. (D) Representative image of TLC and TAG contents in control or *cpl-1* knockdown worms induced by supplementation of glucose or palmitic acid, n=3 independent growths. All data are presented as mean±SEM. ^*^p<0.05; ^**^p<0.01 and ^***^p<0.001 by two tailed student’s t-test.

**Additional file 9: Figure S5.** The efficiency of *cpl-1* knockdown in multiple mutants. (A) Schematic representation of the *cpl-1* gene. RNAi PCR product of *cpl-1* is indicated. Black boxes represent exons and wavy lines represent introns. (B) Real-time PCR analysis of *cpl-1* gene expression in multiple mutants fed with control or *cpl-1* RNAi bacteria. *act-1* was used as reference gene in real-time PCR analysis, n=3 independent growths. (C) The protein expression of CPL-1 in multiple mutants fed with control or *cpl-1* RNAi bacteria, n=3 independent growths. The data in (B) are presented as mean±SEM, ^***^p<0.001 in a two tailed student’s t-test.

**Additional file 10: Figure S6.** The expression of genes related to insulin and TOR signaling in N2 and *cpl-1(qx304)* worms. (A) Real-time PCR analysis of genes related to insulin and TOR signaling in N2 and *cpl-1(qx304)* worms. *act-1* was used as reference gene in real-time PCR analysis, n=3 independent growths. The data are presented as mean±SEM, ^**^p<0.01 and n.s. not significant by two tailed student’s t-test.

**Additional file 11: Figure S7.** The localization of CPL-1 in *C. elegans*. (A) Subcellular localization of CPL-1::mChOint driven by the *cpl-1* promoter. Images of CPL-1::mChOint, and LMP-1-1::GFP and merged images of CPL-1::mChOint with LMP-1::GFP.

**Additional file 12: Figure S8.** The efficiency of tissue-specific *cpl-1* RNAi. (A) Real-time PCR analysis of genes involved in serotonin signaling pathway in tissue-selective *cpl-1* knockdown worms, germline restricted MAH23 (*rrf-1(pk1417) I*), muscle restricted WM118 (*rde-1(ne300) V;nels9 X*) and neurons restricted VH624 (*rhIs13 V; nre-1(hd20) lin-15B(hd126) X*). *act-1* was used as reference gene in real-time PCR analysis, n=3 independent growths. (B) CPL-1::mChOint expression in tissue-selective RNAi strains: intestine restricted VP303 (*rde-1(ne219) V;kzls7*), hypodermis restricted NR222 (*rde-1(ne219) V;kzls9*), germline restricted MAH23 (*rrf-1(pk1417) I*), muscle restricted WM118 (*rde-1(ne300) V;nels9 X*) and neurons restricted VH624 (*rhIs13 V; nre-1(hd20) lin-15B(hd126) X*), during control RNAi or *cpl-1* RNAi treatment. Restricted tissues expressing CPL-1::mChOint were indicated with a white arrow. Int, intestine; Hyp, hypodermis, Ger, germline; Mus, muscle and Neu, neurons. The data in (A) are presented as mean±SEM. n.s. not significant in a two tailed student’s t-test.

**Additional file 13: Figure S9.** Lipid metabolism genes expression in N2 and *cpl-1(qx304)* worms. (A) Real-time PCR analysis of genes involved in lipolysis, fatty acid β-oxidation and lipogenesis in N2 and *cpl-1(qx304)* worms. *act-1* was used as reference gene in real-time PCR analysis, n=3 independent growths. The data are presented as mean±SEM, ^*^p<0.05; ^**^p<0.01; ^***^p<0.001 and n.s. not significant by two tailed student’s t-test.

**Additional file 14: Figure S10.** The physiological index of cathepsin L knockout mice fed with LFD. Male 6-week-old *Ctsl*^*+/+*^ and *Ctsl*^*-/-*^ mice were fed with LFD for 12 weeks. (A) Body weight was recorded every week. (B) White adipose tissues weights were measured at 18-week-old. (C) The food intake per 20 g body weight of mice during 12-week treatment with LFD and HFD. (D and E) At 18 weeks old, the mice metabolic parameters were measured during a 12-h light and 12-h dark cycle and the average for each group in light or dark cycle. (D) Oxygen consumption (VO_2_) and (E) carbon dioxide production (VCO_2_). (F) The contents of serotonin in mice brain fed with LFD. All data are presented as mean±SEM, *n*=10 per group, ^**^p<0.01; ^***^p<0.001 and n.s. not significant in a Nonparametric Mann-Whitney test.

**Additional file 15: Figure S11.** The energy expenditure normalized per animal in *Ctsl*^*+/+*^ and *Ctsl*^*-/-*^ mice fed with HFD. Male 6-week-old *Ctsl*^*+/+*^ and *Ctsl*^*-/-*^ mice were fed with HFD for 12 weeks. At 18 weeks old, the mice metabolic parameters normalized per animal were measured during a 12-h light and 12-h dark cycle and the average for each group in light or dark cycle. (A) Oxygen consumption (VO_2_) and (B) carbon dioxide production (VCO_2_). All data are presented as mean±SEM, n=10 per group, n.s. not significant in a Nonparametric Mann-Whitney test.

**Additional file 16: Table S5.** Primers for quantitative real-time PCR analysis in *C. elegans.*

**Additional file 17: Table S6.** Primers for quantitative real-time PCR analysis in mice.

**Additional file 18: Figure S12.** The data related to specificity of Rabbit Anti-CPL-1 antibody. (A) The 960 bp fragment of *cpl-1*(NM-001269789) was amplified by PCR with the forward primer, 5’-GGCGGATCCGCCAAGCTGTCCCGTCAAAT and the reverse primer, 5’-GGGCTCGAGTTAGACCAATGGATAACTGG. (B) The recombinant plasmid, P*cpl-1*::pET28a, in which the fragment of *cpl-1* was constructed into the plasmid pET28a, was digested by *BamH I* and *XhoΙ* into two fragments 5369 bp and 960 bp. (C) The positive colony of P*cpl-1*::pET28a was detected using the forward primer, 5’-GGCGGATCCGCCAAGCTGTCCCGTCAAAT and the reverse primer, 5’-GGGCTCGAGTTAGACCAATGGATAACTGG. (D) The recombinant protein was expressed in BL21 induced by IPTG. (E) The recombinant protein was purified by Ni-NTA superflow Agarose and eluted by imidazole. (F) The titer of Rabbit Anti-CPL-1 antibody was detected using enzyme-linked immunosorbent assay and the final titer of antibody was about 1:256 000. (G) The specificity of Rabbit Anti-CPL-1 antibody was detected using Western blot in N2 worms fed with control or *cpl-1* RNAi bacteria. The data referred to the paper of ZHAO Lin and BAO Bin published in Journal of Hefei University of Technology, 2018; 41(11):1552-1557.

**Additional file 19.** Individual data values.

## Data Availability

All data generated or analyzed during this study are included in this published article and its supplementary information files. The individual data values are provided in Additional file 19.

## Abbreviations

WAT: White adipose tissues
HFD: High-fat diet
NGM: Nematode Growth Medium
L4 stage: Fourth larval stage
TAG: Triglycerides
TLC: Thin-layer chromatography
LFD: Low-fat diet
*Cstl*^*−/−*^ mice: Cathepsin L-deficient mice
*Cstl*^*+/+*^ mice: Littermate control mice
i.a.: Intracranial
i.p.: Intraperitoneal
PCPA: p-chlorophenylalanine
L1 stage: First larval stage
