# Supplementary material for: Peripheral cathepsin L inhibition induces fat loss in C. elegans and mice through promoting central serotonin synthesis
Source: BMC Biol. 2019 Nov 26;17:93. doi: 10.1186/s12915-019-0719-4 (PMC6880508; doi:10.1186/s12915-019-0719-4)
Supplement: Supplementary file 1 — Additional file 1: Figure S1. The glucose and palmitic acid uptake by E. coli and C. elegans. (A) The concentration of glucose in E. coli seeded on NGM plates with the supplementation of 1 mM or 5 mM glucose. (B) The relative concentration of glucose in C. elegans induced by the supplementation of 1 mM or 5 mM glucose. (C) The relative proportion of palmitic acid content in E. coli seeded on NGM plates with the supplementation of 0.02 mM or 0.2 mM palmitic acid. (D) The relative proportion of palmitic acid content in C. elegans induced by the supplementation of 0.02 mM or 0.2 mM palmitic acid. The data were obtained from 4 growths. All data are presented as mean±SEM, **p<0.01; ***p<0.001 and n.s. not significant by one-way ANOVA. [file 12915_2019_719_MOESM1_ESM.pdf]

Additional file 1: Figure S1

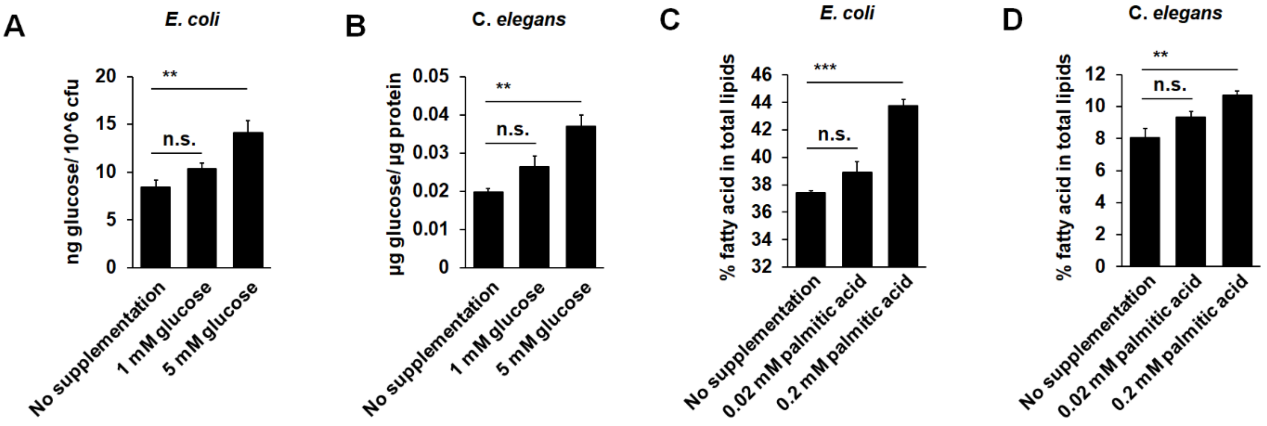

Figure S1. The glucose and palmitic acid uptake by *E. coli* and *C. elegans*.

(A) The concentration of glucose in *E. coli* seeded on NGM plates with the supplementation of 1 mM or 5 mM glucose. (B) The relative concentration of glucose in *C. elegans* induced by the supplementation of 1 mM or 5 mM glucose. (C) The relative proportion of palmitic acid content in *E. coli* seeded on NGM plates with the supplementation of 0.02 mM or 0.2 mM palmitic acid. (D) The relative proportion of palmitic acid content in *C. elegans* induced by the supplementation of 0.02 mM or 0.2 mM palmitic acid. The data were obtained from 4 growths. All data are presented as mean±SEM, $^{**}p<0.01$; $^{***}p<0.001$ and n.s. not significant by one-way ANOVA.
