# Supplementary material for: Peripheral cathepsin L inhibition induces fat loss in C. elegans and mice through promoting central serotonin synthesis
Source: BMC Biol. 2019 Nov 26;17:93. doi: 10.1186/s12915-019-0719-4 (PMC6880508; doi:10.1186/s12915-019-0719-4)
Supplement: Supplementary file 2 — Additional file 2: Table S1. The effect of nutrient supplementation on developmental rate in N2 worms. [file 12915_2019_719_MOESM2_ESM.pdf]

Additional file 2:

Table S1. The effect of nutrient supplementation on developmental rate in N2 worms.

Treatment	Nutrient	Proportion					
		L4 (%)		Adult (%)		Gravid adult (%)	
		mean ±SEM	*p* value	mean ±SEM	*p* value	mean ±SEM	*p* value
No	No	13.67±2.02		81.67±0.89		4.67±1.2	
Nutrient supplementation	1 mM glucose	13.33±1.67	0.9051	78±1.53	0.1062	8.67±1.33	0.0898
	5 mM glucose	12.33±1.33	0.6119	79.67±0.88	0.1841	8±0.58	0.0668
	0.02 mM palmitic acid	12.67±0.89	0.6745	79.67±0.88	0.1841	7.67±0.89	0.1145
	0.2 mM palmitic acid	15.67±0.88	0.4169	77.67±1.67	0.1012	6.67±1.67	0.2508

The worms were grown on NGM plates with nutrients since L4 stage of larvae. At 50 h after synchronization, the numbers of L4, adult and gravid adult worms were visually counted based on the development of the vulva. For each condition, 3 independent experiments were performed and at least 30 worms were scored in each experiment.
