# Supplementary material for: Peripheral cathepsin L inhibition induces fat loss in C. elegans and mice through promoting central serotonin synthesis
Source: BMC Biol. 2019 Nov 26;17:93. doi: 10.1186/s12915-019-0719-4 (PMC6880508; doi:10.1186/s12915-019-0719-4)
Supplement: Supplementary file 3 — Additional file 3: Figure S2. The expression of GFP fluorescence in DHS-3::GFP worms induced by the supplementation of nutrients. (A) Immunoblot and quantification of GFP protein in DHS-3::GFP worms induced by the supplementation of glucose or palmitic acid. The band of GFP protein was counted as the quantification of GFP to ACT-1, n=3 independent growths. (B) Representative images of GFP fluorescence in DHS-3::GFP worms induced by the supplementation of glucose or palmitic acid. Scale bar represents 20 μm. (C) Distribution of the lipid droplet size (% lipid droplets), as measured from images of GFP fluorescence in DHS-3::GFP worms from (B). The data were obtained from 3 independent experiments and 30 worms were imaged and qualified with the level of fluorescence intensity. The data in (A) and (C) are presented as mean±SEM, **p<0.01; ***p<0.001 and n.s. not significant by one-way ANOVA. [file 12915_2019_719_MOESM3_ESM.pdf]

Additional file 3: Figure S2.

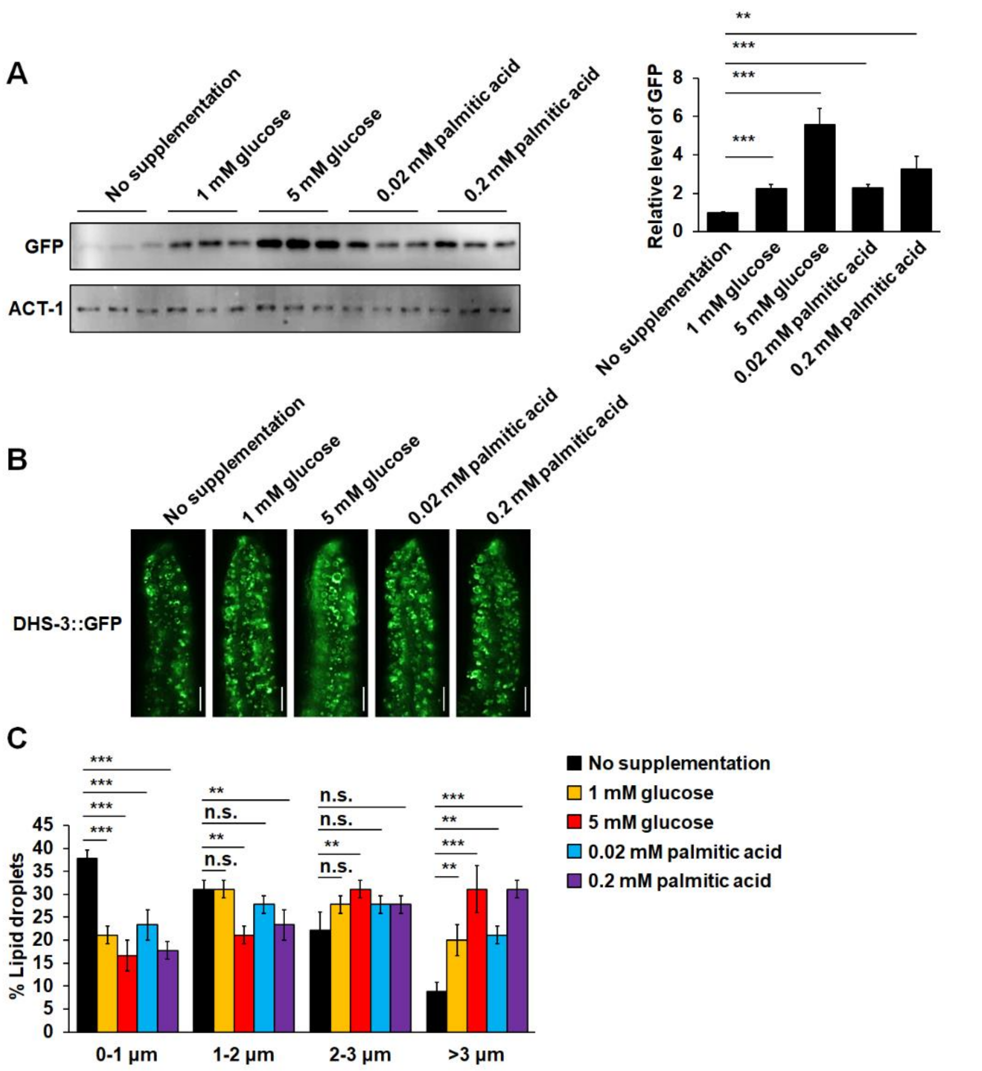

Figure S2. The expression of GFP fluorescence in DHS-3::GFP worms induced by the supplementation of nutrients.

(A) Immunoblot and quantification of GFP protein in DHS-3::GFP worms induced by the supplementation of glucose or palmitic acid. The band of GFP protein was counted as the quantification of GFP to ACT-1, n=3 independent growths. (B) Representative images of GFP fluorescence in DHS-3::GFP worms induced by the supplementation of glucose or palmitic acid. Scale bar represents 20 µm. (C) Distribution of the lipid droplet size (% lipid droplets), as measured from images of GFP fluorescence in DHS-

3::GFP worms from (B). The data were obtained from 3 independent experiments and 30 worms were imaged and qualified with the level of fluorescence intensity. The data in (A) and (C) are presented as mean±SEM, $^{**}p<0.01$; $^{***}p<0.001$ and n.s. not significant by one-way ANOVA.
