# Supplementary material for: Peripheral cathepsin L inhibition induces fat loss in C. elegans and mice through promoting central serotonin synthesis
Source: BMC Biol. 2019 Nov 26;17:93. doi: 10.1186/s12915-019-0719-4 (PMC6880508; doi:10.1186/s12915-019-0719-4)
Supplement: Supplementary file 4 — Additional file 4: Table S2. The change of cathepsin-like gene expression in C. elegans after supplementation of 5 mM glucose. [file 12915_2019_719_MOESM4_ESM.pdf]

Additional file 4:

Table S2. The change of cathepsin-like gene expression in *C. elegans* after supplementation of 5 mM glucose.

Human cathepsin	*C. elegans* cathepsin-like genes	mRNA expression change induced by 5 mM glucose over control	*p* value	Human cathepsin	*C. elegans* cathepsin-like genes	mRNA expression change induced by 5 mM glucose over control	*p* value
cathepsin A	*F13D12.6*	0.74±0.09	0.1021	cathepsin E	*asp-5(F21F8.3)*	0.72±0.17	0.3587
	F32A5.3	0.77±0.09	0.3268		*asp-6(F21F8.7)*	0.98±0.05	0.8909
	F41C3.5	0.91±0.25	0.7445		*asp-9(C11D2.2)*	0.82±0.32	0.546
	K10B2.2	0.77±0.19	0.3198		*asp-10(C15C8.3)*	0.66±0.17	0.051
	Y16B4A.2	0.87±0.17	0.3624		*asp-12(F21F8.4)*	1.67±0.27	0.0478
	Y32F6A.5	0.90±0.03	0.2938		*asp-19(ZK384.6)*	1.15±0.41	0.6204
	K10C2.1	0.69±0.18	0.256	cathepsin F	*R07E3.1*	0.89±0.20	0.6571
	Y40D12A.2	0.77±0.19	0.3247		*F09F10.1*	0.92±0.33	0.824
cathepsin B	*F32H5.1*	1.06±0.15	0.7639		*F41E6.6*	1.03±0.03	0.6503
	F57F5.1	1.42±0.34	0.155	cathepsin H	*K02E7.10*	0.74±0.15	0.1552
	W07B8.1	1.06±0.09	0.5202		*tag-329(C50F4.3)*	0.68±0.18	0.0925
	W07B8.4	1.03±0.27	0.8593	cathepsin L	*Y51A2D.1*	0.86±0.07	0.0732
	Y65B4A.2	0.90±0.14	0.2956		*Y51A2D.8*	0.57±0.13	0.2273
	cpr-1(C52E4.1)	1.38±0.15	0.047		*Y71H2AR.2*	1.06±0.43	0.8467
	cpr-2(F36D3.9)	1.03±0.31	0.921		*cpl-1(T03E6.7)*	2.18±0.43	0.0267
	cpr-3(T10H4.12)	0.64±0.03	0.079	cathepsin S	*C32B5.7*	1.18±0.43	0.5477
	cpr-4(F44C4.3)	1.47±0.06	0.0377		*F15D4.4*	0.68±0.07	0.2207
	cpr-5(W07B8.5)	1.33±0.07	0.039		*Y40H7A.10*	0.93±0.27	0.7997
	cpr-6(C25B8.3)	0.76±0.08	0.0204		*Y71H2AM.25*	1.02±0.19	0.9217
cathepsin D	*asp-1(Y39B6A.20)*	0.89±0.07	0.5417	cathepsin Z	*cpz-1(F32B5.8)*	0.97±0.06	0.7057
	asp-3(H22K11.1)	0.68±0.14	0.2537		*cpz-2(M04G12.2)*	0.78±0.10	0.4888
	asp-4(R12H7.2)	0.90±0.003	0.4969				

N2 worms were grown on NGM plates with or without 5 mM glucose supplementation since L4. Using real-time PCR analysis, the expressions of cathepsin-like genes was

detected in the worms. *act-1* was used as reference gene in real-time PCR analysis, n=3 independent growths. In response to 5 mM glucose supplementation, the highlight gene *cpl-1(T03E6.7)* exhibited the highest enhancement in mRNA expression.
