# Supplementary material for: Peripheral cathepsin L inhibition induces fat loss in C. elegans and mice through promoting central serotonin synthesis
Source: BMC Biol. 2019 Nov 26;17:93. doi: 10.1186/s12915-019-0719-4 (PMC6880508; doi:10.1186/s12915-019-0719-4)
Supplement: Supplementary file 5 — Additional file 5: Figure S3. The expression of cathepsin genes in C. elegans induced by nutrients supplementation. (A) Heatmap of cathepsin genes expression in N2 worms fed with 5 mM glucose at L4 stage for 16 h. See Additional file 4: Table S2 for the data. (B) Real-time PCR analysis of cathepsin genes in N2 worms induced by the supplementation of glucose or palmitic acid. act-1 was used as reference gene in real-time PCR analysis, n=3 independent growths. The data in (B) are presented as mean±SEM, *p<0.05; **p<0.01 and n.s. not significant by one-way ANOVA. [file 12915_2019_719_MOESM5_ESM.pdf]

Additional file 5: Figure S3.

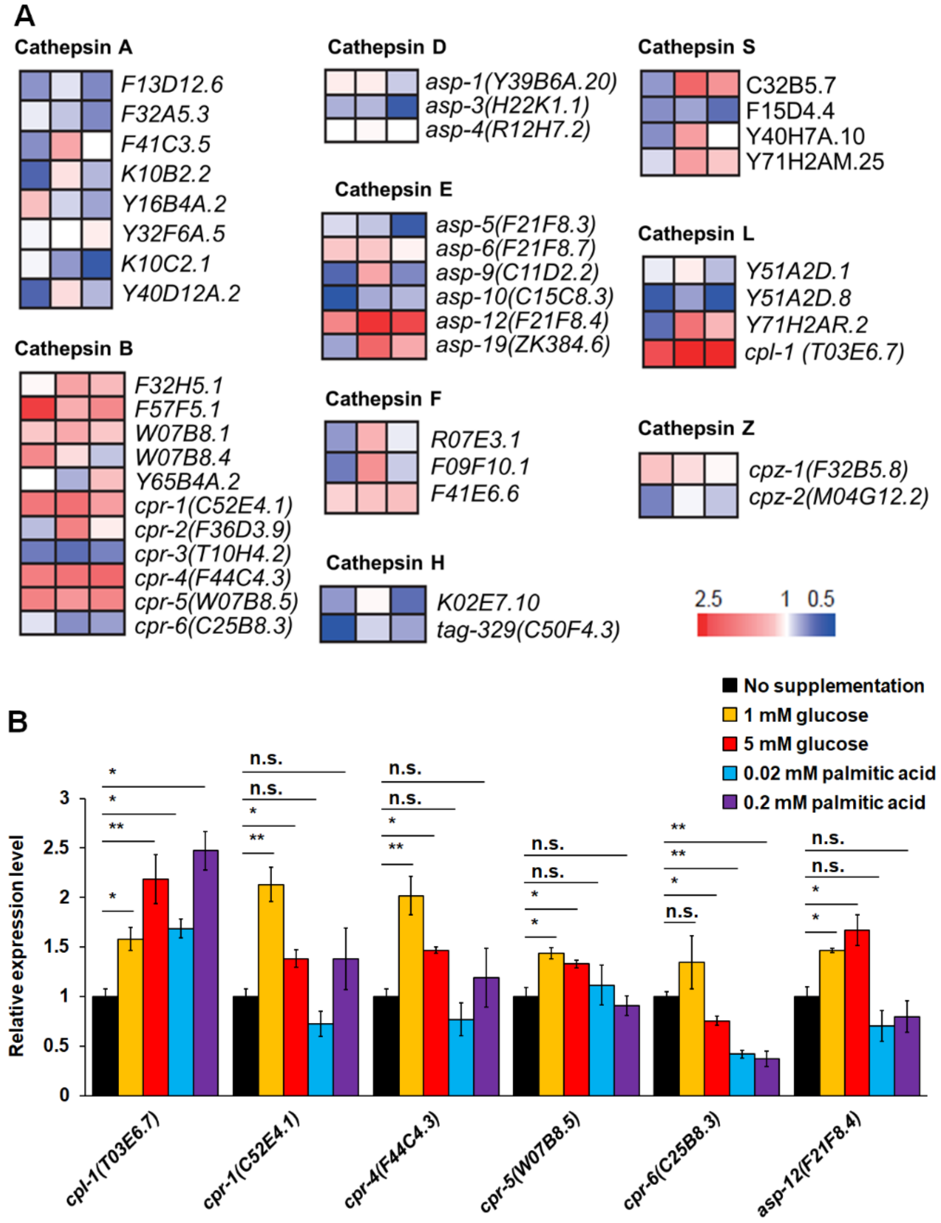

Figure S3. The expression of cathepsin genes in *C. elegans* induced by nutrients supplementation.

(A) Heatmap of cathepsin genes expression in N2 worms fed with 5 mM glucose at L4 stage for 16 h. See Additional file 4: Table S2 for the data. (B) Real-time PCR analysis of cathepsin genes in N2 worms induced by the supplementation of glucose or palmitic acid. *act-1* was used as reference gene in real-time PCR analysis, n=3 independent

growths. The data in (B) are presented as mean±SEM, $^{*}p<0.05$; $^{**}p<0.01$ and n.s. not significant by one-way ANOVA.
