# Supplementary material for: Peripheral cathepsin L inhibition induces fat loss in C. elegans and mice through promoting central serotonin synthesis
Source: BMC Biol. 2019 Nov 26;17:93. doi: 10.1186/s12915-019-0719-4 (PMC6880508; doi:10.1186/s12915-019-0719-4)
Supplement: Supplementary file 6 — Additional file 6: Table S3. The effect of cpl-1 mutation on developmental rate in C. elegans. [file 12915_2019_719_MOESM6_ESM.pdf]

Additional file 6:

Table S3. The effect of *cpl-1* mutation on developmental rate in *C. elegans*.

Strains	Proportion					
	L4 (%)		Adult (%)		Gravid adult (%)	
	mean ± SEM	*p* value	mean ± SEM	*p* value	mean ± SEM	*p* value
N2	13.67 ±2.02		81.67 ±0.89		4.67 ±1.2	
cpl-1(ok360)	17 ±2.65	0.3739	77.33 ±3.18	0.2594	5.67 ±0.67	0.5072
cpl-1(qx304)	17 ±1.15	0.2263	77.67 ±2.02	0.0866	6.33 ±0.88	0.3262
cpl-1(yq89)	16.33 ±1.76	0.3772	78.67 ±1.2	0.1145	5 ±0.58	0.8149

L1 larva of N2 worms and *cpl-1* mutants were placed to the plates with OP50 bacteria maintaining at 20 °C. At 50 h after synchronization, the numbers of L4, adult and gravid adult worms were visually counted based on the development of the vulva. For each condition, 3 independent experiments were performed and at least 30 worms were scored in each experiment.
