# Supplementary material for: Peripheral cathepsin L inhibition induces fat loss in C. elegans and mice through promoting central serotonin synthesis
Source: BMC Biol. 2019 Nov 26;17:93. doi: 10.1186/s12915-019-0719-4 (PMC6880508; doi:10.1186/s12915-019-0719-4)
Supplement: Supplementary file 7 — Additional file 7: Table S4. The effect of cpl-1 RNAi on developmental rate in multiple mutants. [file 12915_2019_719_MOESM7_ESM.pdf]

Additional file 7:

Table S4. The effect of *cpl-1* RNAi on developmental rate in multiple mutants.

Strains	Treatment L1-adult	Proportion					
		L4 (%)		Adult (%)		Gravid adult (%)	
		mean ± SEM	*p* value	mean ± SEM	*p* value	mean ± SEM	*p* value
N2	Control RNAi	2.33 ±0.88		63.33 ±4.33		34.33 ±3.48	
	cpl-1 RNAi	3.33 ±0.88	0.4676	66 ±1.53	0.5928	30.67 ±2.19	0.4227
VP303	Control RNAi	5 ±1.53		71 ±2.52		24 ±2.31	
	cpl-1 RNAi	5 ±1.53	1	71.33 ±3.48	0.9419	23.67 ±2.4	0.9252
NR222	Control RNAi	5.67 ±1.86		71 ±1		23.33 ±2.6	
	cpl-1 RNAi	4 ±2.08	0.5823	73 ±4.93	0.7114	23 ±2.89	0.9358
MAH23	Control RNAi	5.33 ±0.88		70.67 ±4.33		24 ±3.79	
	cpl-1 RNAi	4.33 ±1.45	0.5879	73.33 ±5.36	0.7178	22.33 ±4.18	0.7822
WM118	Control RNAi	3.67 ±2.19		71.67 ±2.19		24.67 ±4.37	
	cpl-1 RNAi	4 ±2.08	0.9174	73.33 ±4.84	0.7694	22.67 ±0.105	0.7398
VH624	Control RNAi	4 ±2		71.67 ±5.04		24.33 ±3.18	
	cpl-1 RNAi	5 ±1.53	0.7114	72.33 ±3.71	0.9204	22.67 ±2.33	0.6943
daf-2 (e1370)	Control RNAi	42 ±3.06		56.67 ±3.48		1.33 ±0.88	
	cpl-1 RNAi	48.67 ±4.91	0.3132	49.67 ±4.1	0.2627	1.67 ±1.2	0.834
rict-1 (ft7)	Control RNAi	46.33 ±3.18		50.67 ±4.06		3 ±1.53	
	cpl-1 RNAi	41 ±8.96	0.6049	57 ±8.39	0.5339	2 ±1.15	0.6291
tph-1 (mg280)	Control RNAi	3.33 ±0.88		72 ±2.52		24.67 ±3.28	
	cpl-1 RNAi	2.33 ±0.88	0.4676	72.33 ±2.4	0.9283	25.33 ±2.6	0.8813
mod-1 (ok103)	Control RNAi	26.33 ±2.73		72 ±2		1.67 ±0.88	
	cpl-1 RNAi	27.67 ±1.45	0.6885	70 ±2.08	0.5265	2.33 ±1.33	0.6981
ser-6 (tm2146)	Control RNAi	11 ±2.08		84 ±1		5 ±1.15	
	cpl-1 RNAi	9.33 ±2.19	0.6102	81.33 ±1.76	0.2588	9.33 ±3.84	0.3411
mod-1(ok103; ser-6 (tm2146)	Control RNAi	26 ±3.79		72 ±2.65		2 ±1.15	
	cpl-1 RNAi	24.33 ±3.18	0.753	73.33 ±2.96	0.754	2.33 ±0.88	0.8298

L1 larva were placed to the plates with RNAi bacteria and incubated at 20 °C. At 60 h after synchronization, the numbers of L4, adult and gravid adult worms were visually counted based on the development of the vulva. For each condition, 3 independent experiments were performed and at least 30 worms were scored in each experiment.
