# Supplementary material for: Peripheral cathepsin L inhibition induces fat loss in C. elegans and mice through promoting central serotonin synthesis
Source: BMC Biol. 2019 Nov 26;17:93. doi: 10.1186/s12915-019-0719-4 (PMC6880508; doi:10.1186/s12915-019-0719-4)
Supplement: Supplementary file 8 — Additional file 8: Figure S4. Functional inactivation of CPL-1 decreased the fat accumulation in C. elegans. (A) Representative images and quantification of DHS-3::GFP fluorescence in N2 and cpl-1(qx304) worms induced by the supplementation of glucose or palmitic acid. The data were obtained from 3 independent experiments and 30 worms were imaged and qualified. (B) Representative image of TLC and TAG contents in N2 and cpl-1(qx304) worms induced by the supplementation of glucose or palmitic acid, n=3 independent growths. (C) Representative images and quantification of DHS-3::GFP fluorescence in control or cpl-1 knockdown worms induced by supplementation of glucose or palmitic acid. The data were obtained from 3 independent experiments and 30 worms were imaged and qualified. (D) Representative image of TLC and TAG contents in control or cpl-1 knockdown worms induced by supplementation of glucose or palmitic acid, n=3 independent growths. All data are presented as mean±SEM. *p<0.05; **p<0.01 and ***p<0.001 by two tailed student’s t-test. [file 12915_2019_719_MOESM8_ESM.pdf]

Additional file 8: Figure S4

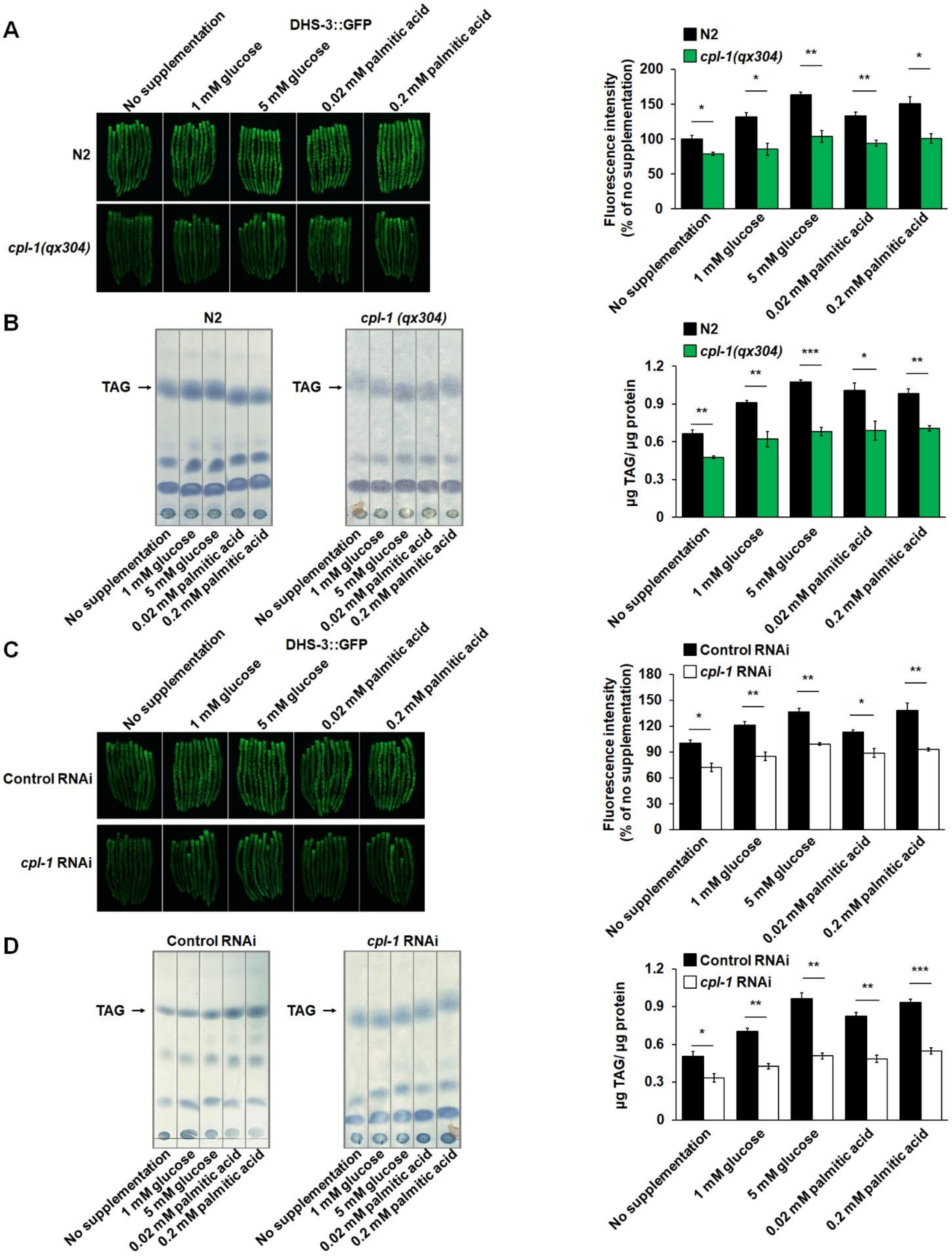

Figure S4. Functional inactivation of CPL-1 decreased the fat accumulation in *C. elegans*.

(A) Representative images and quantification of DHS-3::GFP fluorescence in N2 and *cpl-1(qx304)* worms induced by the supplementation of glucose or palmitic acid. The data were obtained from 3 independent experiments and 30 worms were imaged and qualified. (B) Representative image of TLC and TAG contents in N2 and *cpl-1(qx304)* worms induced by the supplementation of glucose or palmitic acid, n=3 independent growths. (C) Representative images and quantification of DHS-3::GFP fluorescence in control or *cpl-1* knockdown worms induced by supplementation of glucose or palmitic acid. The data were obtained from 3 independent experiments and 30 worms were imaged and qualified. (D) Representative image of TLC and TAG contents in control or *cpl-1* knockdown worms induced by supplementation of glucose or palmitic acid, n=3 independent growths. All data are presented as mean±SEM. $^{*}p<0.05$; $^{**}p<0.01$ and $^{***}p<0.001$ by two tailed student's t-test.
