# Supplementary material for: Peripheral cathepsin L inhibition induces fat loss in C. elegans and mice through promoting central serotonin synthesis
Source: BMC Biol. 2019 Nov 26;17:93. doi: 10.1186/s12915-019-0719-4 (PMC6880508; doi:10.1186/s12915-019-0719-4)
Supplement: Supplementary file 9 — Additional file 9: Figure S5. The efficiency of cpl-1 knockdown in multiple mutants. (A) Schematic representation of the cpl-1 gene. RNAi PCR product of cpl-1 is indicated. Black boxes represent exons and wavy lines represent introns. (B) Real-time PCR analysis of cpl-1 gene expression in multiple mutants fed with control or cpl-1 RNAi bacteria. act-1 was used as reference gene in real-time PCR analysis, n=3 independent growths. (C) The protein expression of CPL-1 in multiple mutants fed with control or cpl-1 RNAi bacteria, n=3 independent growths. The data in (B) are presented as mean±SEM, ***p<0.001 in a two tailed student’s t-test. [file 12915_2019_719_MOESM9_ESM.pdf]

Additional file 9: Figure S5.

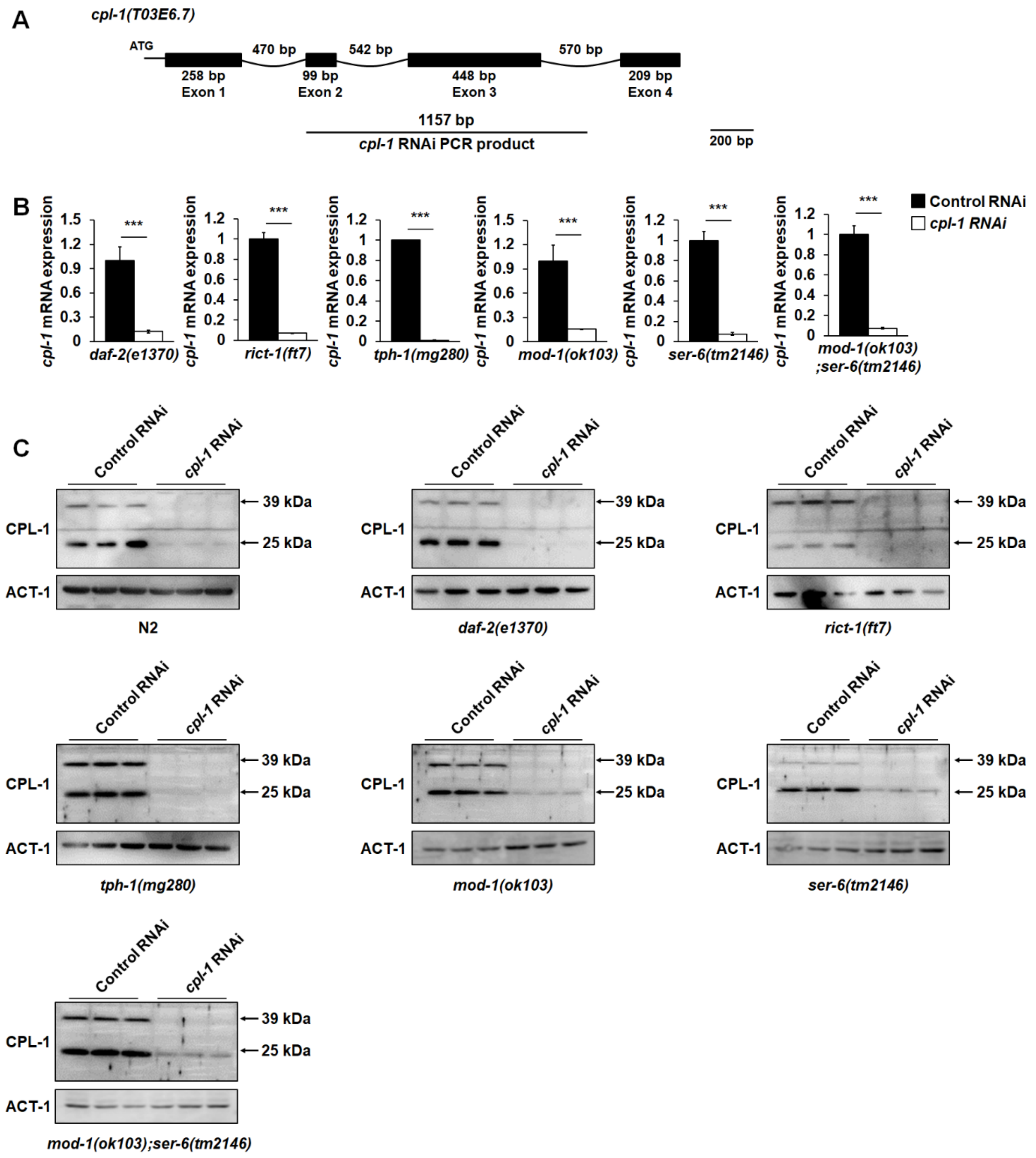

Figure S5. The efficiency of *cpl-1* knockdown in multiple mutants.

(A) Schematic representation of the *cpl-1* gene. RNAi PCR product of *cpl-1* is indicated. Black boxes represent exons and wavy lines represent introns. (B) Real-time PCR analysis of *cpl-1* gene expression in multiple mutants fed with control or *cpl-1* RNAi

bacteria. *act-1* was used as reference gene in real-time PCR analysis, n=3 independent growths. (C) The protein expression of CPL-1 in multiple mutants fed with control or *cpl-1* RNAi bacteria, n=3 independent growths. The data in (B) are presented as mean±SEM, $^{***}p<0.001$ in a two tailed student's t-test.
