# Supplementary material for: Peripheral cathepsin L inhibition induces fat loss in C. elegans and mice through promoting central serotonin synthesis
Source: BMC Biol. 2019 Nov 26;17:93. doi: 10.1186/s12915-019-0719-4 (PMC6880508; doi:10.1186/s12915-019-0719-4)
Supplement: Supplementary file 10 — Additional file 10: Figure S6. The expression of genes related to insulin and TOR signaling in N2 and cpl-1(qx304) worms. (A) Real-time PCR analysis of genes related to insulin and TOR signaling in N2 and cpl-1(qx304) worms. act-1 was used as reference gene in real-time PCR analysis, n=3 independent growths. The data are presented as mean±SEM, **p<0.01 and n.s. not significant by two tailed student’s t-test. [file 12915_2019_719_MOESM10_ESM.pdf]

Additional file 10: Figure S6

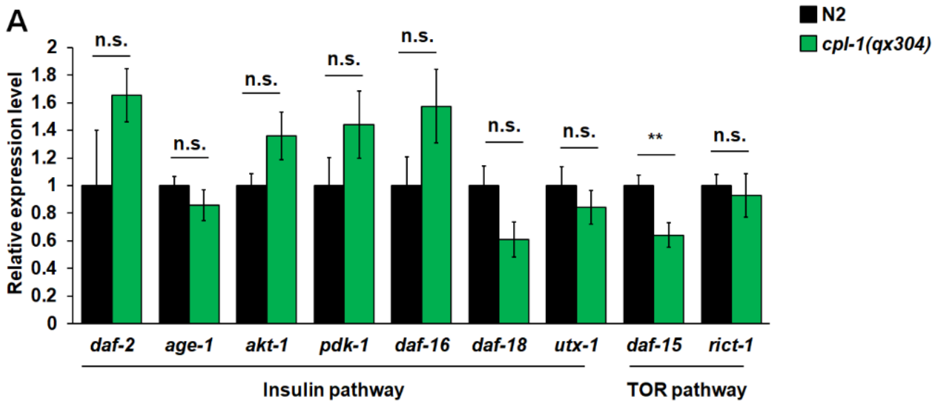

Figure S6. The expression of genes related to insulin and TOR signaling in N2 and *cpl-1(qx304)* worms.

(A) Real-time PCR analysis of genes related to insulin and TOR signaling in N2 and *cpl-1(qx304)* worms. *act-1* was used as reference gene in real-time PCR analysis, n=3 independent growths. The data are presented as mean±SEM, $^{**}p<0.01$ and n.s. not significant by two tailed student's t-test.
