# Supplementary material for: Peripheral cathepsin L inhibition induces fat loss in C. elegans and mice through promoting central serotonin synthesis
Source: BMC Biol. 2019 Nov 26;17:93. doi: 10.1186/s12915-019-0719-4 (PMC6880508; doi:10.1186/s12915-019-0719-4)
Supplement: Supplementary file 11 — Additional file 11: Figure S7. The localization of CPL-1 in C. elegans. (A) Subcellular localization of CPL-1::mChOint driven by the cpl-1 promoter. Images of CPL-1::mChOint, and LMP-1-1::GFP and merged images of CPL-1::mChOint with LMP-1::GFP. [file 12915_2019_719_MOESM11_ESM.pdf]

Additional file 11: Figure S7.

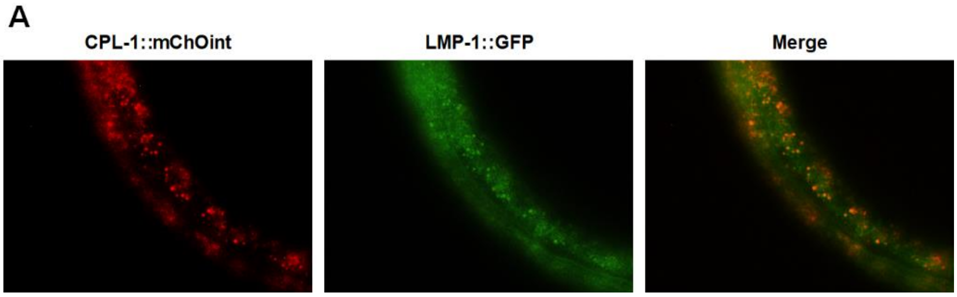

Figure S7. The localization of CPL-1 in *C. elegans*.

(A) Subcellular localization of CPL-1::mChOint driven by the *cpl-1* promoter. Images of CPL-1::mChOint, and LMP-1-1::GFP and merged images of CPL-1::mChOint with LMP-1::GFP.
