# Supplementary material for: Peripheral cathepsin L inhibition induces fat loss in C. elegans and mice through promoting central serotonin synthesis
Source: BMC Biol. 2019 Nov 26;17:93. doi: 10.1186/s12915-019-0719-4 (PMC6880508; doi:10.1186/s12915-019-0719-4)
Supplement: Supplementary file 12 — Additional file 12: Figure S8. The efficiency of tissue-specific cpl-1 RNAi. (A) Real-time PCR analysis of genes involved in serotonin signaling pathway in tissue-selective cpl-1 knockdown worms, germline restricted MAH23 (rrf-1(pk1417) I), muscle restricted WM118 (rde-1(ne300) V;nels9 X) and neurons restricted VH624 (rhIs13 V; nre-1(hd20) lin-15B(hd126) X). act-1 was used as reference gene in real-time PCR analysis, n=3 independent growths. (B) CPL-1::mChOint expression in tissue-selective RNAi strains: intestine restricted VP303 (rde-1(ne219) V;kzls7), hypodermis restricted NR222 (rde-1(ne219) V;kzls9), germline restricted MAH23 (rrf-1(pk1417) I), muscle restricted WM118 (rde-1(ne300) V;nels9 X) and neurons restricted VH624 (rhIs13 V; nre-1(hd20) lin-15B(hd126) X), during control RNAi or cpl-1 RNAi treatment. Restricted tissues expressing CPL-1::mChOint were indicated with a white arrow. Int, intestine; Hyp, hypodermis, Ger, germline; Mus, muscle and Neu, neurons. The data in (A) are presented as mean±SEM. n.s. not significant in a two tailed student’s t-test. [file 12915_2019_719_MOESM12_ESM.pdf]

Additional file 12: Figure S8

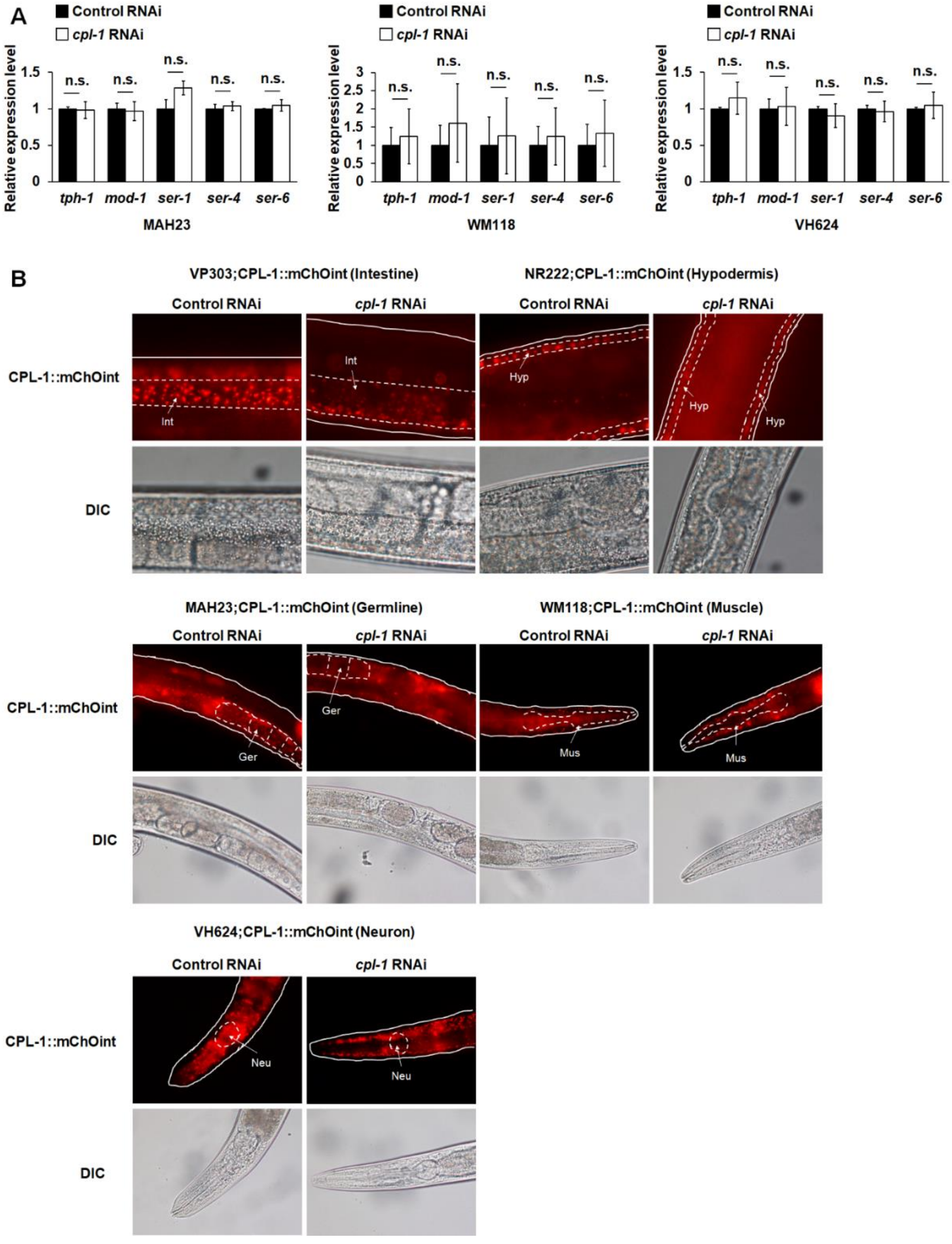

Figure S8. The efficiency of tissue-specific *cpl-1* RNAi.

(A) Real-time PCR analysis of genes involved in serotonin signaling pathway in tissue-selective *cpl-1* knockdown worms, germline restricted MAH23 (*rrf-1(pk1417) I*), muscle restricted WM118 (*rde-1(ne300) V;nels9 X*) and neurons restricted VH624 (*rhIs13 V; nre-1(hd20) lin-15B(hd126) X*). *act-1* was used as reference gene in real-time PCR analysis, n=3 independent growths. (B) CPL-1::mChOint expression in tissue-selective RNAi strains: intestine restricted VP303 (*rde-1(ne219) V;kzls7*), hypodermis restricted NR222 (*rde-1(ne219) V;kzls9*), germline restricted MAH23 (*rrf-1(pk1417) I*), muscle restricted WM118 (*rde-1(ne300) V;nels9 X*) and neurons restricted VH624 (*rhIs13 V; nre-1(hd20) lin-15B(hd126) X*), during control RNAi or *cpl-1* RNAi treatment. Restricted tissues expressing CPL-1::mChOint were indicated with a white arrow. Int, intestine; Hyp, hypodermis, Ger, germline; Mus, muscle and Neu, neurons. The data in (A) are presented as mean±SEM. n.s. not significant in a two tailed student's t-test.
