# Supplementary material for: Peripheral cathepsin L inhibition induces fat loss in C. elegans and mice through promoting central serotonin synthesis
Source: BMC Biol. 2019 Nov 26;17:93. doi: 10.1186/s12915-019-0719-4 (PMC6880508; doi:10.1186/s12915-019-0719-4)
Supplement: Supplementary file 13 — Additional file 13: Figure S9. Lipid metabolism genes expression in N2 and cpl-1(qx304) worms. (A) Real-time PCR analysis of genes involved in lipolysis, fatty acid β-oxidation and lipogenesis in N2 and cpl-1(qx304) worms. act-1 was used as reference gene in real-time PCR analysis, n=3 independent growths. The data are presented as mean±SEM, *p<0.05; **p<0.01; ***p<0.001 and n.s. not significant by two tailed student’s t-test. [file 12915_2019_719_MOESM13_ESM.pdf]

Additional file 13: Figure S9.

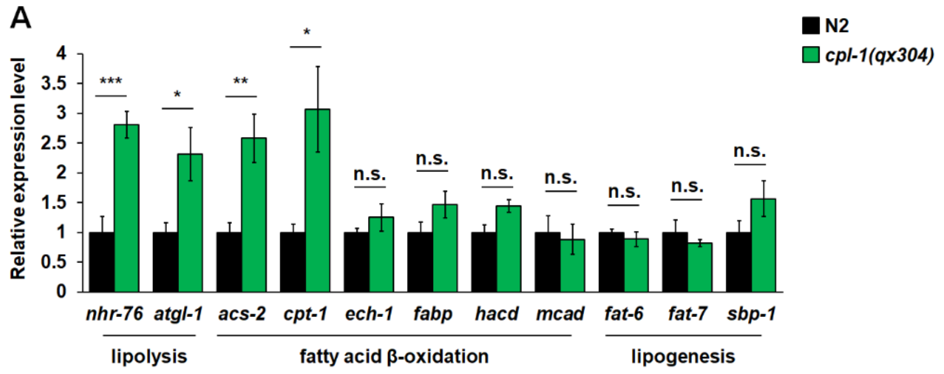

Figure S9. Lipid metabolism genes expression in N2 and *cpl-1(qx304)* worms.

(A) Real-time PCR analysis of genes involved in lipolysis, fatty acid β-oxidation and lipogenesis in N2 and *cpl-1(qx304)* worms. *act-1* was used as reference gene in real-time PCR analysis, n=3 independent growths. The data are presented as mean±SEM, $^{*}p<0.05$; $^{**}p<0.01$; $^{***}p<0.001$ and n.s. not significant by two tailed student's t-test.
