# Supplementary material for: Peripheral cathepsin L inhibition induces fat loss in C. elegans and mice through promoting central serotonin synthesis
Source: BMC Biol. 2019 Nov 26;17:93. doi: 10.1186/s12915-019-0719-4 (PMC6880508; doi:10.1186/s12915-019-0719-4)
Supplement: Supplementary file 14 — Additional file 14: Figure S10. The physiological index of cathepsin L knockout mice fed with LFD. Male 6-week-old Ctsl+/+ and Ctsl-/- mice were fed with LFD for 12 weeks. (A) Body weight was recorded every week. (B) White adipose tissues weights were measured at 18-week-old. (C) The food intake per 20 g body weight of mice during 12-week treatment with LFD and HFD. (D and E) At 18 weeks old, the mice metabolic parameters were measured during a 12-h light and 12-h dark cycle and the average for each group in light or dark cycle. (D) Oxygen consumption (VO2) and (E) carbon dioxide production (VCO2). (F) The contents of serotonin in mice brain fed with LFD. All data are presented as mean±SEM, n=10 per group, **p<0.01; ***p<0.001 and n.s. not significant in a Nonparametric Mann-Whitney test. [file 12915_2019_719_MOESM14_ESM.pdf]

Additional file 14: Figure S10.

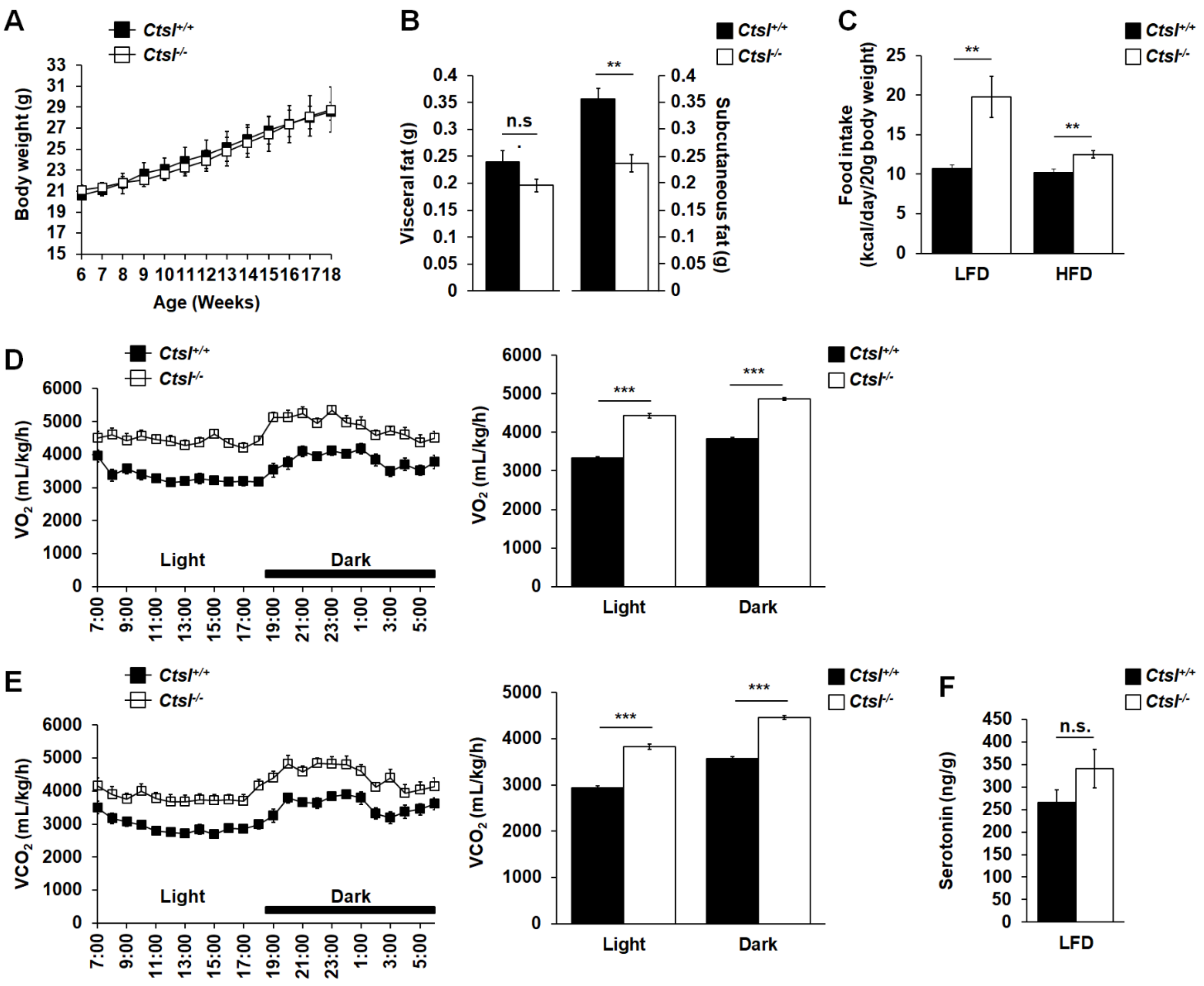

Figure S10. The physiological index of cathepsin L knockout mice fed with LFD.

Male 6-week-old *Ctsl*$^{+/+}$ and *Ctsl*$^{-/-}$ mice were fed with LFD for 12 weeks. (A) Body weight was recorded every week. (B) White adipose tissues weights were measured at 18-week-old. (C) The food intake per 20 g body weight of mice during 12-week treatment with LFD and HFD. (D and E) At 18 weeks old, the mice metabolic parameters were measured during a 12-h light and 12-h dark cycle and the average for each group in light or dark cycle. (D) Oxygen consumption (VO_2) and (E) carbon dioxide production (VCO_2). (F) The contents of serotonin in mice brain fed with LFD.

All data are presented as mean±SEM, n=10 per group, $^{**}p<0.01$; $^{***}p<0.001$ and n.s. not significant in a Nonparametric Mann-Whitney test.
