# Supplementary material for: Peripheral cathepsin L inhibition induces fat loss in C. elegans and mice through promoting central serotonin synthesis
Source: BMC Biol. 2019 Nov 26;17:93. doi: 10.1186/s12915-019-0719-4 (PMC6880508; doi:10.1186/s12915-019-0719-4)
Supplement: Supplementary file 15 — Additional file 15: Figure S11. The energy expenditure normalized per animal in Ctsl+/+ and Ctsl-/- mice fed with HFD. Male 6-week-old Ctsl+/+ and Ctsl-/- mice were fed with HFD for 12 weeks. At 18 weeks old, the mice metabolic parameters normalized per animal were measured during a 12-h light and 12-h dark cycle and the average for each group in light or dark cycle. (A) Oxygen consumption (VO2) and (B) carbon dioxide production (VCO2). All data are presented as mean±SEM, n=10 per group, n.s. not significant in a Nonparametric Mann-Whitney test. [file 12915_2019_719_MOESM15_ESM.pdf]

Additional file 15: Figure S11.

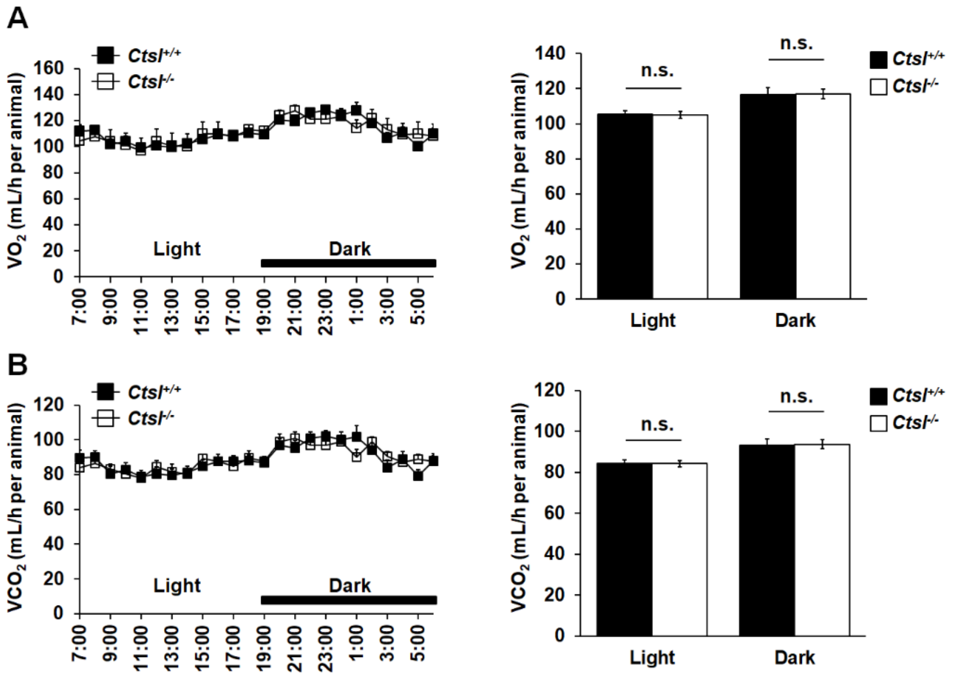

Figure S11. The energy expenditure normalized per animal in *Ctsl*$^{+/+}$ and *Ctsl*$^{-/-}$ mice fed with HFD.

Male 6-week-old *Ctsl*$^{+/+}$ and *Ctsl*$^{-/-}$ mice were fed with HFD for 12 weeks. At 18 weeks old, the mice metabolic parameters normalized per animal were measured during a 12-h light and 12-h dark cycle and the average for each group in light or dark cycle. (A) Oxygen consumption (VO_2) and (B) carbon dioxide production (VCO_2). All data are presented as mean±SEM, n=10 per group, n.s. not significant in a Nonparametric Mann-Whitney test.
