# Supplementary material for: Peripheral cathepsin L inhibition induces fat loss in C. elegans and mice through promoting central serotonin synthesis
Source: BMC Biol. 2019 Nov 26;17:93. doi: 10.1186/s12915-019-0719-4 (PMC6880508; doi:10.1186/s12915-019-0719-4)
Supplement: Supplementary file 16 — Additional file 16: Table S5. Primers for quantitative real-time PCR analysis in C. elegans. [file 12915_2019_719_MOESM16_ESM.pdf]

Additional file 16:

Table S5. Primers for quantitative real-time PCR analysis in *C. elegans*

C. elegans genes	Forward sequences (5' to 3')	Reverse sequences (5' to 3')
act-1	GCCGGAGACGACGCTCCACGCG	GCCTCGTCTCCGACGTACGAGTC
F13D12.6	TCGTTCAGCGAGTTCTCGGC	GTACGCCGACTGCATCTCCA
F32A5.3	GTGCCTACCATGGACGTGTC	GACTACGTAGGAGATGGCTC
F41C3.5	GGCTGATGTTCCATGCCTCA	GGGATATCTGCAGTGACAAG
K10B2.2	ATGCAGCTACCACAGTTCCG	CCAGCATGGGAAGAGTGTAG
Y16B4A.2	GTTTCTCCACGTCAGCCATG	TCACTGTCAA AGGATCAGGAC
Y32F6A.5	GAGTATCGTACTCGTCCGTTC	CAACTCAACTTCCGCGCTGT
K10C2.1	ACTCCTGG AACAAGGCTG CT	AGACCGCTTT GGACACCTAC
Y40D12A.2	GATGCCATGGA ATGACCCAG	TCC CAGCTGCAAC TGAACAG
F32H5.1	ACCCATGGTC TCTGTACTGG	GGTACAACATCTGTGCCGTG
F57F5.1	TCGGACAATC TGCATACGCC	TACTCCGGAG GAGTCTACGT
W07B8.1	GTATTCCTAC CGGAGGATCC	AATGTGACCT ACCCAGCGTG
W07B8.4	TCAATTGCTCCATGCGGCG A	CCCGATTCC ATATGACCAGG
Y65B4A.2	CCACTGAAGG CCTTGACCTA	TTGA CCTGTCGTGTGGAGTC
cpr-1(C52E4.1)	TTCGGAGTCTCTGCCTACGC	GAATGCAGCCTCAACTGGTCC
cpr-2(F36D3.9)	GGCTCGCTGTCAACTCTTGG	CGGCAATCCAGCGACAATACG
cpr-3(T10H4.12)	GATGAAGTTCAAAGTGATGGATGTG	AAAGTGTCGGGGAGTGGTTCT
cpr-4(F44C4.3)	GCCTACACCGCTGACAAGCAC	GTGAATGCGGCCTCGACTGG
cpr-5(W07B8.5)	TGCGGCGAGACTGTGAA	GGGTGGCGTAGTTGTTCTTG
cpr-6(C25B8.3)	TTCGGATGTAACGGAGGAGA	TGGGAATGGGTATGGCTTG
asp-1(Y39B6A.20)	CGCTCCACTC TTCACTGTCT	CCATCGACAC CGTCAACTGC
asp-3(H22K11.1)	ATCTTCGCCA ACTCCGCCAT	ACACCGATCC GAACCACTAC
asp-4(R12H7.2)	GCGGTTCTCG GAGTTCAACC	CAACCGTAACCCAGACTCTG
asp-5(F21F8.3)	GCTGTCGACC ATGTCGTTCC	TGAGCACAAG GGATCTGCCA
asp-6(F21F8.7)	GAATCCTTGA CCAGCCACTC	TACGGAGCCA TTGACACCAC
asp-9(C11D2.2)	CATCCTGACTCCAGCGAATG	CATGGATCCTTGGTGATCCATT
asp-10(C15C8.3)	CTGGGTCATACAGTCAAGCTC	AGTCTTGCCAAGGCGTGAGT
asp-12(F21F8.4)	GGAATTCTCG GGCTTGCCTT	CGATCAA CCTCTGTTCA CAGTC

asp-19(ZK384.6)	AACTGGTGGGTGATTGGCTC	TCAGCACTGAATACGGGCTG
R07E3.1	GGAATGGCGGT TATTCAGCC	CGAGGTAATCGGACTGCATG
R09F10.1	GGAGAATCAGCCTACTGGATTG	AGTGAACAGC TGCGGTCTTG
F41E6.6	AA GACATCGTGC ACATCAGA	TCACGACGAC TCCATCACTG
K02E7.10	GCAAGAGCTC ACATCACCAC	CACCAAGG AAGAATGCGGGA
tag-329	AGCTGAAGCT GCTCTGACAG	ATGCAATGGA GGCGATCCAG
Y51A2D.1	TTCGCGGTCA ACCAGTTCTC	ACTGCTTGGC AAGACCCGTA
Y51A2D.8	CAGGTGCTCA CCGAGTGGAA	GAG GACGATTGTA GAAGAGC
Y71H2AR.2	ATGCATCCCA CGCATTTGCG	TCTGAGCAGCAGCTTATTGATTG
cpl-1(T03E6.7)	GGTCCCAGATGAGGTTGACTGGC	GCGAGCGTGTTGTCCTTCGAGGG
C32B5.7	GAAGGAGTCG TTGGACCTGT	TC CATGTACGCC ATCGCCAA
F15D4.4	CGACGAT GGCTACATAT CAGG	GATTGCAGTG GGAATGGCTG
Y40H7A.10	TAGTGGTGGA GGTCTGCAGA	GTGAGCCAGTTGAAGCTCTC
Y71H2AM.25	CAAGGCTACGA ACGGATCAC	AGGGATGTGA AGAGCAACCG
cpz-1(F32B5.8)	GGGCGGTGTCTACAAGTATGC	GGTCGCATTTTCCGTCTCTC
cpz-2(M04G12.2)	GAGCACGCTA AGATCCAAGG	GAGCACGCTA AGATCCAAGG
daf-2	CCAAGCCCTCACCTACTTCC	CTCTGAGGGCTGACACAAGG
age-1	GCTGCTCCGTGCAGAGATTG	CACGGAGGTAAGCTTCCATC
akt-1	CGAACTTATCATGGCTGGTGA	CCCAATCGCTGAGTTGGATC
pdk-1	CCGAGTACCCGTATCACCAG	GTGCAGGACTGGTGGCTTGA
daf-16	CAGTTCCGTGGCCAGTTCCA	GGTCTGCTGCTGTTGAGAGG
daf-18	GGACGGGAAGATTGGTCATGT	CGTCTCCGATGTACGGCTGA
utx-1	GCACACGCTAAAGGGTGAAGCT	CGGTGGAGAGCTGGCTCGCTTA
daf-15	GTCGAAGCAGGAATGCCCGA	CGTGGCATTTCGACAGTCGC
rict-1	GCCACGTCTCGAATAAGCACT	GGGCCACCGTTGATGATCTC
tph-1	TGACGCTGCCGATTCTCCAG	GCATGTTGCAACTCGCCAGC
mod-1	GGATGTGTGGATGCTTGGATGC	TTACGCTGTTCTGACAACGGGA
ser-6	CTGCTCCCTACTTCTGGCTGTC	GACGGGAGAGATGTGGGTTGAC
ser-1	TCACACGCGACGAGACTCGT	CCATTGGTTCTGGCGACTGG
ser-4	CCACAGCGACTGCCTTCTAC	TTACGGTACGTCGGTCGCGT
nhr-76	CACCGGCTTAAAGATGCGAC	TCCATCTGTCGGAACACGCT
atgl-1	CTGGCCTAGATCGACCGATG	CGTATGCATGGAGCCAATCC

cpt-1	TATGAACCAGCATCAGCTCG	GACGATCGACTCCTTGCCC
fabp	TTCTCGAGTTGAGAGATCGG	ATTCTGGCTCTCCGATCCG
acs-2	GCAGCCTCGCTCTACACTCT	GACTCCTGCAAATGCACATGC
ech-1	CGTCGGAGCT GGATTCATGG	TCGTTCAACG CCTGCCTGGT
mcad	TCGACAACAAGGTGCGCTC	GAATTTGCGAGGTTCCCTCG
hacd	CAGGACTTATGGATACTCCAC	GGCATGCGGAGTGCTCCG
fat-6	TCGGACTCTACCAGCTCATC	TGAGCTCCGGCGGTTATTCC
fat-7	TTGGTGCATCAACAGCGCTG	ACCAACGGCTACAACTGTGG
sbp-1	GATTGCTCGCTGGAAGTGCG	CCGAGTGCTAGTTCCATCCG
