# Supplementary material for: Peripheral cathepsin L inhibition induces fat loss in C. elegans and mice through promoting central serotonin synthesis
Source: BMC Biol. 2019 Nov 26;17:93. doi: 10.1186/s12915-019-0719-4 (PMC6880508; doi:10.1186/s12915-019-0719-4)
Supplement: Supplementary file 17 — Additional file 17: Table S6. Primers for quantitative real-time PCR analysis in mice. [file 12915_2019_719_MOESM17_ESM.pdf]

Additional file 17:

Table S6. Primers for quantitative real-time PCR analysis in mice.

Mice genes	Forward sequences (5' to 3')	Reverse sequences (5' to 3')
β-Actin	CATCCGTAAAGACCTCTATGCCAAC	ATGGAGCCACCGATCCACA
Atgl	TGACCATCTGCCTTCCAGA	TGTAGGTGGCGCAAGACA
Hsl-1	GCGCTGGAGGAGTGTTTTT	CCGCTCTCCAGTTGAACC
Acsl1	TGGGGTTGGAAATCATCAGCC	CACAGCATTACACACTCTACAACGG
Peci	CCCTTCTGGGACTATTTGATGCT	TGGCTGAATGGAGTATGAAACG
Acox1	TAACTTCCTCACTCGAAGCCA	AGTTCCATGACCCATCTCTGTC
Cpt1a	CTATGCGCTACTCGCTGAAGG	GGCTTTCGACCCGAGAAGA
Tph2	GCAAGACAGCGGTAGTGTTCA	CAGTCCACGAAGATTTCGATT
