# Supplementary material for: Peripheral cathepsin L inhibition induces fat loss in C. elegans and mice through promoting central serotonin synthesis
Source: BMC Biol. 2019 Nov 26;17:93. doi: 10.1186/s12915-019-0719-4 (PMC6880508; doi:10.1186/s12915-019-0719-4)
Supplement: Supplementary file 18 — Additional file 18: Figure S12. The data related to specificity of Rabbit Anti-CPL-1 antibody. (A) The 960 bp fragment of cpl-1(NM-001269789) was amplified by PCR with the forward primer, 5’-GGCGGATCCGCCAAGCTGTCCCGTCAAAT and the reverse primer, 5’-GGGCTCGAGTTAGACCAATGGATAACTGG. (B) The recombinant plasmid, Pcpl-1::pET28a, in which the fragment of cpl-1 was constructed into the plasmid pET28a, was digested by BamH I and XhoΙ into two fragments 5369 bp and 960 bp. (C) The positive colony of Pcpl-1::pET28a was detected using the forward primer, 5’-GGCGGATCCGCCAAGCTGTCCCGTCAAAT and the reverse primer, 5’-GGGCTCGAGTTAGACCAATGGATAACTGG. (D) The recombinant protein was expressed in BL21 induced by IPTG. (E) The recombinant protein was purified by Ni-NTA superflow Agarose and eluted by imidazole. (F) The titer of Rabbit Anti-CPL-1 antibody was detected using enzyme-linked immunosorbent assay and the final titer of antibody was about 1:256 000. (G) The specificity of Rabbit Anti-CPL-1 antibody was detected using Western blot in N2 worms fed with control or cpl-1 RNAi bacteria. The data referred to the paper of ZHAO Lin and BAO Bin published in Journal of Hefei University of Technology, 2018; 41(11):1552-1557. [file 12915_2019_719_MOESM18_ESM.pdf]

# Additional file 18: Figure S12.

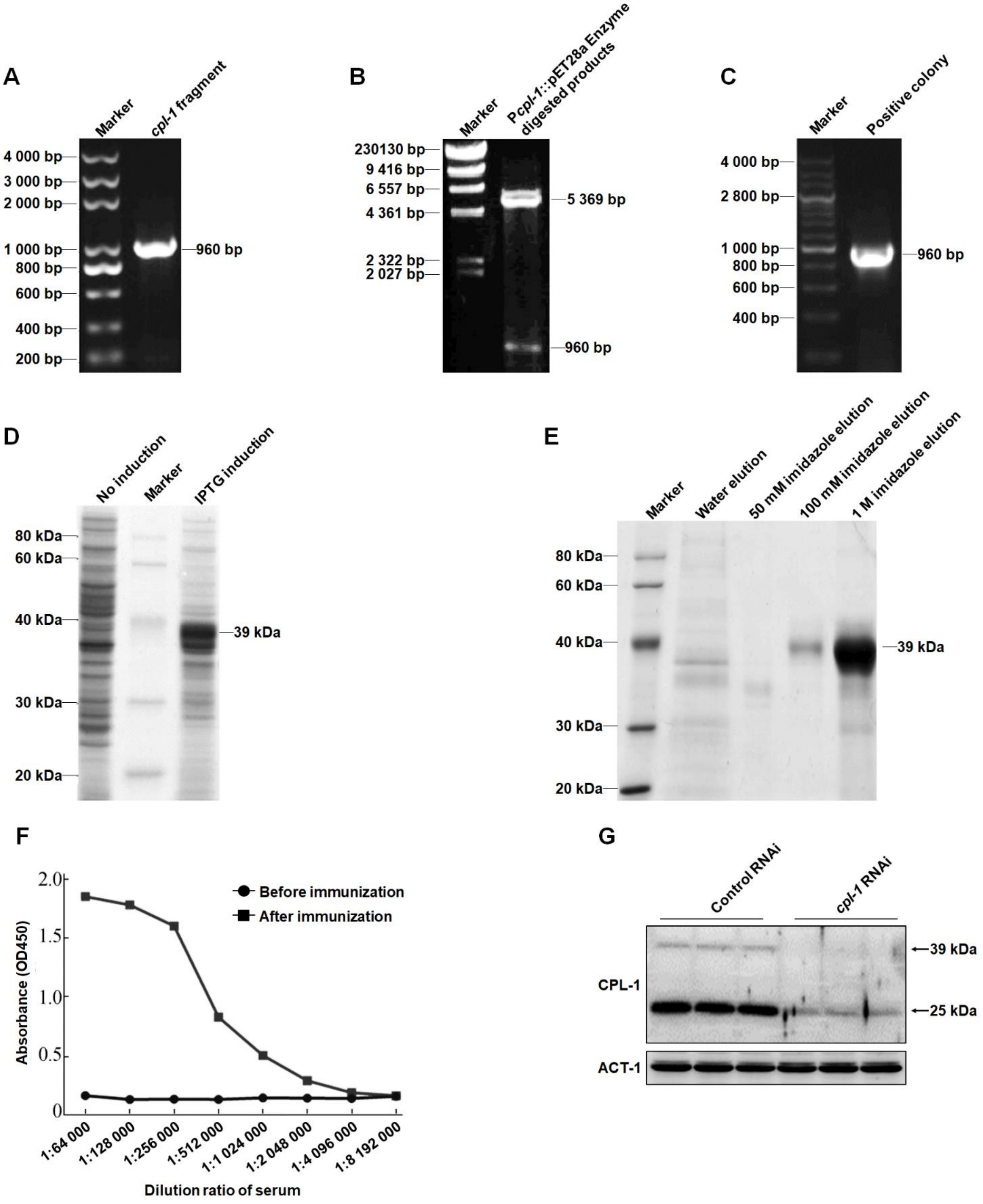


**Figure S12. The data related to specificity of Rabbit Anti-CPL-1 antibody.**

(A) The 960 bp fragment of *cpl-1*(NM-001269789) was amplified by PCR with the

forward primer, 5’-GGCGGATCCGCCAAGCTGTCCCGTCAAAT and the reverse primer, 5’-GGGCTCGAGTTAGACCAATGGATAACTGG. (B) The recombinant plasmid, P*cpl-1*::pET28a, in which the fragment of *cpl-1* was constructed into the plasmid pET28a, was digested by *BamH I* and *XhoI* into two fragments 5369 bp and 960 bp. (C) The positive colony of P*cpl-1*::pET28a was detected using the forward primer, 5’-GGCGGATCCGCCAAGCTGTCCCGTCAAAT and the reverse primer, 5’-GGGCTCGAGTTAGACCAATGGATAACTGG. (D) The recombinant protein was expressed in BL21 induced by IPTG. (E) The recombinant protein was purified by Ni-NTA superflow Agarose and eluted by imidazole. (F) The titer of Rabbit Anti-CPL-1 antibody was detected using enzyme-linked immunosorbent assay and the final titer of antibody was about 1:256 000. (G) The specificity of Rabbit Anti-CPL-1 antibody was detected using Western blot in N2 worms fed with control or *cpl-1* RNAi bacteria. The data referred to the paper of ZHAO Lin and BAO Bin published in Journal of Hefei University of Technology, 2018; 41(11):1552-1557.
